# Teleocidin-producing genotype of *Streptomyces clavuligerus* ATCC 27064

**DOI:** 10.1007/s00253-022-11805-5

**Published:** 2022-02-09

**Authors:** Petra Pivk Lukančič, Tjaša Drčar, Robert Bruccoleri, Martin Črnugelj, Peter Mrak

**Affiliations:** 1 Novartis Technical Operations, MS&T Antiinfectives, Mengeš, Slovenia; 2 Congenomics, LLC, Glastonbury, CT USA; 3 Sandoz Development Center Slovenia, Physical Analytics Department, Ljubljana, Slovenia

**Keywords:** Clavulanic acid, Teleocidin, *Streptomyces clavuligerus*, Lyngbyatoxin, Genome, Secondary metabolite, Toxin

## Abstract

**Abstract:**

*Streptomyces clavuligerus* is an industrially important producer of clavulanic acid (CA), a β-lactamase inhibitor which is used together with amoxicillin in one of the most widely prescribed antibacterial medicines, the co-amoxiclav. In a mid-eighties ATCC vial of *S*. *clavuligerus* ATCC 27064 culture, we have found a new genotype, which was apparently lost from the subsequent ATCC collection stocks, and has remained obscure to the scientific community. Most importantly, this genotype harbors teleocidin (lyngbyatoxin) biosynthetic genes, which are located on an enigmatic 138 kb chromosomal region and support accumulation of significant amounts of these highly toxic, tumor-promoting secondary metabolites in cultures of *S*. *clavuligerus*. While this genomic region is completely absent from all published sequences for *S*. *clavuligerus* ATCC strain, at least one of the industrial strains for commercial production of CA, originating from ATCC 27064, retained the genetic potential for production of teleocidins. The origin of teleocidin biosynthetic cluster can now be traced back to early *S*. *clavuligerus* stocks at the ATCC. Our work provides a genome sequence and a deposited monoisolate of this genotype. Given the scale of industrial use of *S*. *clavuligerus* world-wide and toxicity of teleocidins, we also discuss the environmental and safety implications and provide a method of abolishing teleocidin production without affecting productivity of CA.

**Key points:**

• *Early stocks of S. clavuligerus ATCC 27064 produce toxic teleocidins*

• *Teleocidin biosynthetic genes were found within a distinct S. clavuligerus genotype*

• *The genotype has been passed on to some industrial clavulanic acid producer strains*

**Supplementary Information:**

The online version contains supplementary material available at 10.1007/s00253-022-11805-5.

## Introduction

*Streptomyces clavuligerus* is the producer of clavulanic acid (CA), a medically and industrially important β-lactamase inhibitor which is annually produced and consumed in more than 1000 metric tons worldwide (Bortone et al. 2021; Paradkar 2013; Saudagar et al. 2008). Consequently, hundreds of studies on the biology and genetics of *S*. *clavuligerus* ATCC 27064 (NRRL 3585), the original CA-producing strain, have been reported in the last decades (Lopez-Agudelo et al. 2021), including several independent genome sequencing projects from the first wave of next generation sequencing (NCBI BioProject accession: PRJNA19249; PRJNA28551 and PRJNA42475). The availability of the genome sequence provided an insight into peculiarities of the genome, the incredible wealth of secondary metabolite gene clusters encoded within the main 6.7 Mb chromosome, the large 1.8 Mb linear plasmid (Medema et al. 2010), and several other smaller genomic elements (Song et al. 2010). Numerous genome-supported studies in transcriptomics, proteomics, metabolomics, and regulatory networks, as well as genome mining approaches were done using these genome sequences, which consequently hold a “golden standard” status in the community.

In our laboratory, we have independently sequenced and compared genomes of *S*. *clavuligerus* ATCC 27064 cultures, which were obtained from ATCC (American Type Culture Collection) on 3 occasions: in 1991, 1996, and 2003. Although the bulk of the sequence between the genomes was identical, we have found additional ~ 138 kb of DNA sequence in the main chromosome of *S*. *clavuligerus* ATCC 27064 culture, acquired in 1991 (labeled “Dec. 15. 1986” from the ATTC). Similarity searches for this fragment within the published genomes of *S*. *clavuligerus* ATCC 27064 (PRJNA19249, PRJNA28551, and PRJNA42475), and even across entire public nucleotide databases, gave no clue about its origin until a recent publication of the genome sequence of an industrial *S*. *clavuligerus* strain F613-1 (Cao et al. 2016). This genome provided the first similarity hit for the mysterious 138 kb sequence. A subsequent study by the same group has provided some insight into the plasticity of the genome of *S*. *clavuligerus*, however gave no explanation for the origin of the 138 kb region (Li et al. 2018).

**Fig. 1:**
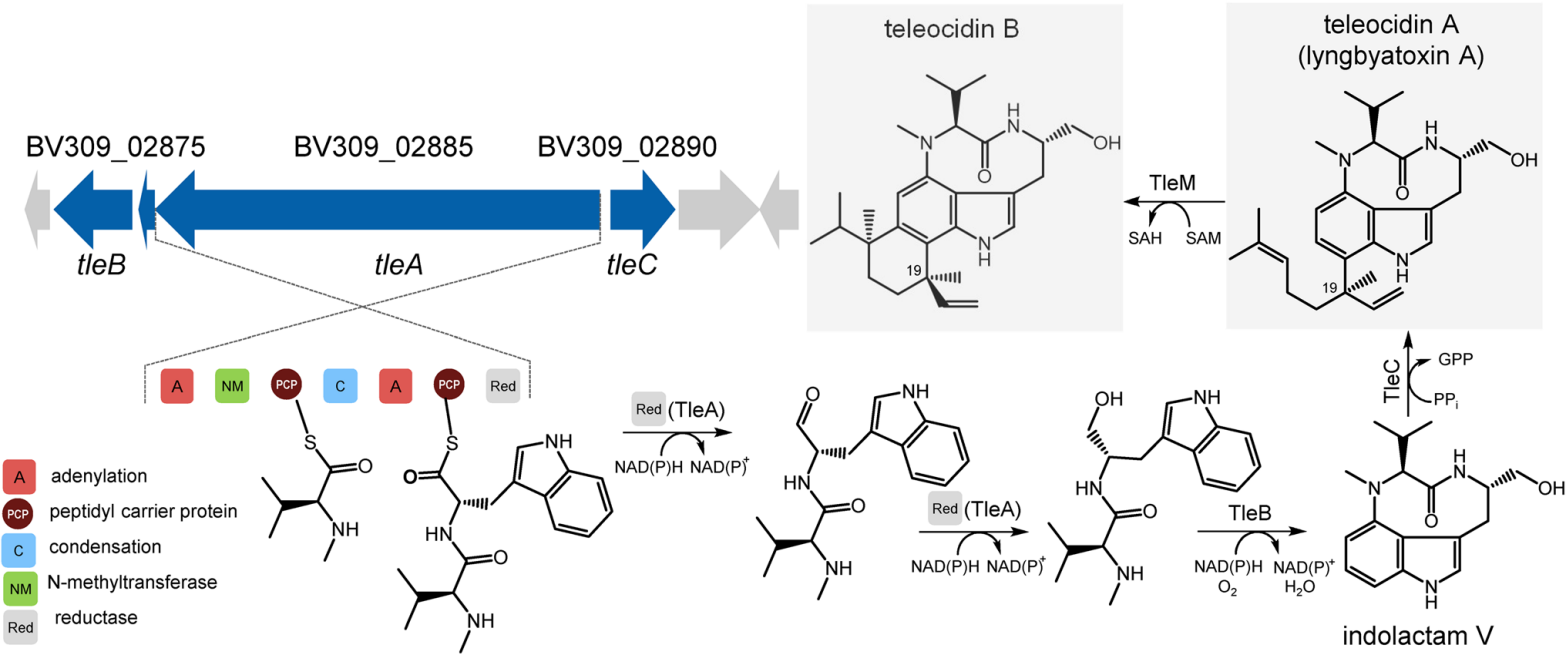
The teleocidin biosynthetic gene cluster found in *S*. *clavuligerus* K4567 and the biosynthetic pathway (Abe 2018) for the major products teleocidin A1 and teleocidin B1. The compounds are produced together with their minor diastereoisomers at position 19 (teleocidins A2 and B2). For simplicity, the latter are not shown throughout this article

Here, we report isolation of a second, stable genotype strain of *S. clavuligerus* ATCC 27064 (hereafter referred to as K4567) from the mixed genotype culture found in the vials prepared by ATCC in 1986. This genotype contains the 138 kb region in the main chromosome and displays distinct phenotypic features. Most notably, a secondary-metabolite gene cluster found within this new genetic region encodes biosynthesis of teleocidins; highly toxic, tumor-promoting indolactam-terpenoid secondary metabolites, which act through activation of protein kinase C (Fujiki et al. 1981, 1984). The major product, teleocidin A (lyngbyatoxin A; Fig. 1; Takashima and Sakai 1960; Cardellina et al. 1979), was isolated and its structure confirmed by NMR. In addition, HRMS analysis indicated the presence of teleocidin B (Fujiki et al. 1979), and other putative congeners. With genetic modifications, we have confirmed identity and activity of the *S*. *clavuligerus* teleocidin gene cluster *in vivo*. We have also found that deletion of the teleocidin gene cluster, or even of the complete 138 kb region unique to the *S*. *clavuligerus* K4567, does not affect clavulanic acid productivity potential.

## Materials and methods

### Strains and culture conditions

*S*. *clavuligerus* ATCC 27064 was obtained from ATCC collection on 3 separate occasions; in 1991, 1996, and 2003. The vial obtained from ATCC in 1991 was labeled “Dec. 15. 1986.” ISP2 agar medium (Passari et al. 2018) was used for plating, and the plates were incubated at 28 °C for 14 days. Seed cultures were inoculated from a patch of confluent culture on ISP2 plates (2 × 2 cm) into 250 mL Erlenmeyer flasks with 50 mL of SNK-t medium (Drčar et al. 2011). Cultures were then incubated on a rotary shaker at 28 °C and 260 rpm for 40 h. Then, 0.3 mL of seed culture was transferred to 100 mL Erlenmeyer flask containing 15 mL of main culture media. The latter was described by Ortiz et al. (2007), and has the following composition (in g per L): glycerol, 10.0; soybean meal, 20.0; soybean oil, 23.0; K_2_HPO_4_, 1.2; MnCl_2_·4H_2_O, 0.001; FeSO_4_·7H_2_O, 0.001; ZnSO_4_·7H_2_O, 0.001, and MOPS buffer, 21 (100 mM). pH was adjusted to 6.8 with NaOH prior to sterilization (121 °C, 15 min) and measured between 6.8 and 6.9 after sterilization. Cultures were incubated on a rotary shaker at 25 °C, 260 rpm (2.5 cm throw) for 5 days and sampled periodically. Where indicated, 1.5 mL cultures were grown at 25 °C and 260 rpm (2.5 cm throw) in 24-well microtiter plates (MTP) equipped with luminescent dissolved oxygen sensors (Deep-well OxoDish, Presens, Germany). The luminescence lifetime is proportional to dissolved oxygen levels and is detected non-invasively through the transparent bottom, placed on an optical detection array (SDR Sensor Dish Reader, Presens, Germany). Multiple parallel flasks/wells were inoculated so that each one flask/well was discarded after sampling while others were left incubating until the next sampling. Rapid phenotype screening was performed in 96 deep-well MTP, containing 500 µL of main culture medium per well and inoculated with a single colony. MTPs were incubated on a rotary shaker at 26 °C and 500 rpm (1.25 cm throw) for 5 days and then sampled. *S*. *clavuligerus* K4567 was deposited at DSMZ under DSM33546.

### Genome sequencing and assembly

Mycelium from 72-h-old TSB cultures of *S*. *clavuligerus* was used for genomic DNA extraction. The extraction was performed according to Nikodinovic et al. (2003). Genome sequencing was performed on the PacBio Sequel platform with sequencing library preparation performed using the SMRTbell template prep kit and SequelTM Binding Kit 2.0 (Pacific Biosciences, Menlo Park, CA USA). HGAP software (Pacific Biosciences) was used to assemble the noisy PacBio reads through a multi-step process. First, an all against all comparison of the reads is used to identify regions of likely identity as well as repetitive regions. Alignments are seeded in the unique sequences and they can span over the repetitive regions. Next, the reads are corrected using a consensus algorithm based on these alignments. The corrected reads are then combinatorially compared, and overlapping regions are used to assemble the genome. The assembly for *S*. *clavuligerus* K4567 resulted in four supercontigs composed of the chromosome (6,881,717 bp) and several plasmids (pSCL1, 10,431 bp; pSCL2, 147,182 bp and pSCL4, 1,761,737 bp). The genome is deposited under NCBI BioProject accession PRJNA360257, genome version JADANO000000000. Gene calling was performed internally using Prodigal software (Hyatt et al. 2010) and natural products pathways were annotated using antiSMASH 3.0 (Weber et al. 2015). The genome was annotated at NCBI using PGAP (Tatusova et al. 2016). The two approaches resulted in a similar outcome; specifically, we have found no differences in gene calling within the 138 kb region in question. Sequence similarity was studied with BLAST (Altschul et al. 1990) and sequence alignments were performed with MUSCLE (Edgar 2004) and LASTZ (Harris 2007).

### Molecular methods

Colony PCR was performed using Q5® High-Fidelity DNA Polymerase (New England Biolabs) and Mastercycler Vapo protect (Eppendorf). After initial denaturation (5 min at 98 °C), 35 amplification cycles (30 s at 95 °C; 20 s at 62 °C; 90 s at 72 °C) were performed and completed with 7 min final elongation at 72 °C. PCR primer pairs are listed in Supplemental Table S1.

The deletion mutants were prepared with double cross-over recombination, guided by an editing template. A classic positive/negative selection approach with a conjugative suicide vector was used (Drčar et al. 2011). The flanking fragments (editing template) for deletion of the *tleA* gene were amplified from the K4567 genome using primer pair 1 for the left and primer pair 2 for the right flank (Supplemental Table S1). The editing template for deletion of the complete 138 kb region was assembled from fragments amplified from the genome using primer pair 3 for the left flank and primer pair 4 for the right flank (Supplemental Table S1).

The editing templates were cloned into a conjugative suicide vector with positive/negative selection markers (*aac*(*3*)*IV* and *codA*) (Drčar et al. 2011). The completed vector (Supplemental Fig. S1) was introduced into *S*. *clavuligerus* by conjugal transfer from *Escherichia coli* ET12567 pUZ8002 using well-established methods (Kieser et al. 2000). Exoconjugants were selected for resistance to apramycin and subjected to selection for loss of the *codA* gene in presence of 5-fluorocytosine (Drčar et al. 2011). After loss of all markers, colonies were screened by PCR to distinguish between w.t. revertants and deletion mutants. A 1273-bp-long PCR product was obtained exclusively with the *tleA* deletion mutants when using the primer pair 5 while primer pair 6 gave a 1337 bp product only with the w.t. genotype (Supplemental Table S1; Supplemental Figs. S2-S3). Similarly, primer pair 7 gave a 978 bp PCR product exclusively for the 138 kb region deletion mutant while the use of primer pair 8 gave a 1005 bp PCR product with the w.t. genotype (Supplemental Table S1; Supplemental Figs. S2-S3).

### Analytical and statistical methods

Analysis of teleocidins in *S. clavuligerus* cultures was performed with LC-UVMS. The fermentation broth was extracted with acetonitrile (1:4) and centrifuged. Clear supernatant (5 μL) was injected onto a Phenomenex Kinetex C18 column (100 × 2.1 mm, 1.7 µm) with a flow of 0.4 mL min^−1^, column temperature 60 °C, and UV diode array multi-wavelength detection. Mobile phase A (MPA) was 0.1% (v/v) formic acid in 1% acetonitrile, mobile phase B (MPB) was 0.1% (v/v) formic acid in 80% acetonitrile. The gradient profile for the method was started at 70% MPB and after 1.2 min progressed linearly to 100% MPB in 5.3 min, with 3 min hold time and final re-equilibration for 1.1 min. LCQ Fleet MS (Thermo Scientific) equipped with HESI source was used for detection. Positive ionization (source voltage 4 kV, vaporizer temperature 125 °C capillary temperature 300 °C, sheath gas 42 AU, auxiliary gas 5 AU) and full-scan monitoring with m/z range 100–500 was used. The compounds were detected primarily in the protonated form [M + H]^+^; teleocidin A (lyngbyatoxin A), *m/z* = 438; teleocidin B, *m/z* = 452. UV signal (λ = 290–310 nm) was used for quantification of teleocidin A by comparison of chromatographic peak against the authentic reference teleocidin A1 (BIA-T1429, Bioaustralis, AU). HRMS was performed following chromatography as above, and detecting on a Q Exactive MS instrument (Thermo Scientific) equipped with HESI ion source operating in positive mode with the same settings as above.

Clavulanic acid was analyzed in culture supernatants with a modified HPLC method (Agilent Technologies 2007), and quantified by comparison to authentic pure lithium clavulanate. Phosphoric acid/water/acetonitrile (0.05%/75%/25%) was used for mobile phase on Zorbax SB-Aq column (Agilent, USA) with detection at 220 nm. Statistical analysis of the data and their presentation was performed with GraphPad Prism 9.

### Isolation of teleocidin A

The fermentation broth was extracted with a mixture of *n*-butanol:acetone:diethyl ether (1:1.5:1). After evaporation of diethyl ether and acetone, the remaining extract formed 3 separate phases. The top *n*-butanol phase was separated and mixed with water (3:1) to facilitate evaporation of solvents at milder conditions by formation of *n*-butanol/water azeotrope. Solvents were evaporated to concentrate the material to ~ 20 g L^−1^. This was subjected to preparative chromatography: 4 mL was injected onto a Thermo Syncronis C18 column (250 × 21.2 mm, 5 µm, flow: 26 mL min^−1^, column temperature: 60 °C). Mobile phase A (MPA) was 1% acetonitrile and mobile phase B (MPB) was 80% acetonitrile. The method was isocratic for 12 min, followed with a gradient from 70% MPB to 100% MPB in 13 min. A 10 min holding time (100% MPB) was completed with a final re-equilibration for 6 min. Ten-milliliter fractions were collected and analyzed. Fractions containing teleocidin A were pooled and solvents evaporated. This material (~ 60 mg) was dissolved in acetonitrile and injected again for a polishing run. The purest fractions were pooled and solvents evaporated. Thirty-eight milligrams of this material was dissolved in 0.7 mL of CDCl_3_ (D, 99.8%, Merck) and characterized by ^1^H and ^13^C NMR on a Bruker Avance III spectrometer operating at 500 and 150 MHz for ^1^H- and ^13^C-NMR, respectively. The spectrometer was equipped with 5 mm BBO, Z-gradient probe. Spectra were acquired and processed using Bruker TopSpin software (ver. 3.1). Chemical shifts (δ) are expressed in ppm with reference to residual solvent signal (7.27 ppm and 77.0 ppm for ^1^H- and ^13^C-NMR, respectively).

## Results

### Genomic differences between cultures of *S. clavuligerus* ATCC 27064

We first performed a preliminary genome comparison between cultures from three different *S*. *clavuligerus* ATCC 27064 vials obtained from ATCC in 1991, 1996, and 2003 (data not shown). While the genomes of strains from 1996 and 2003 were found highly similar to the published ATCC 27064 genomes, we have found additional genomic variants in the *S*. *clavuligerus* ATCC 27064 culture from the earliest ATCC vial (labeled “Dec. 15. 1986” by the ATCC; Supplemental Fig. S4). One of the variants contained a mysterious additional 138 kb region within the main chromosome. A monoisolate obtained from this culture after two sub-plating rounds, the strain K4567, was sequenced again (NCBI BioProject accession: PRJNA360257). A detailed investigation of the assembly at the insertion junctions (positions 441,384 and 579,552) revealed a multitude of raw reads spanning across the insertion boundaries, which excludes the possibility of stand-alone genetic element or contamination with heterogeneous DNA (Supplemental Fig. S5). No similarity to this fragment could be found with the deposited *S*. *clavuligerus* ATCC 27064 genomes (PRJNA19249, PRJNA28551, and PRJNA42475). Upon publication of the genome of *S*. *clavuligerus* F613-1 (PRJNA329150; Cao et al. 2016), a whole-genome alignment was made between the genome of *S*. *clavuligerus* K4567 and F613-1 (Supplemental Fig. S6). A 99.9% identity was found over the region of 138 kb between positions 441,669 and 579,837, relative to latter genome.

**Fig. 2:**
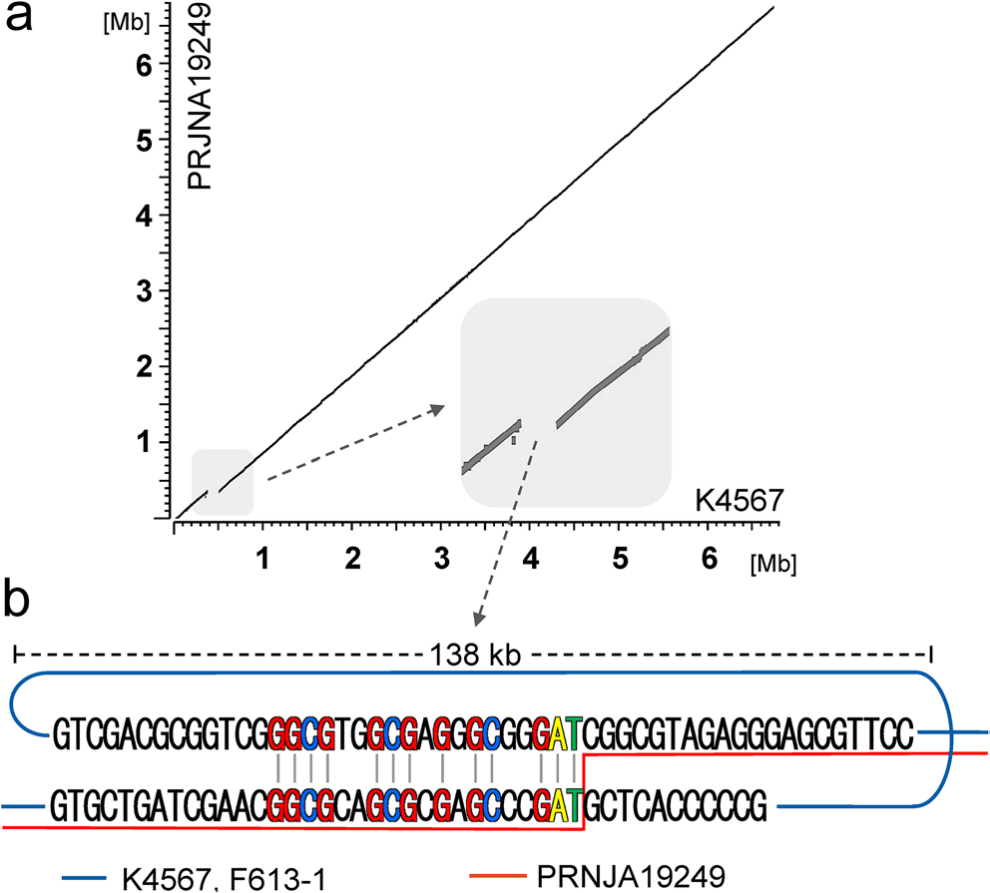
**a** Alignment of main chromosomes of *S*. *clavuligerus* strains K4567 (PRJNA360257) vs. contemporary ATCC 27064 (PRJNA19249). **b** Detailed view of the joint sequence. Aligned sequences of left and right border of the 138 kb region in K4567 and F613-1. The joint sequence of ATCC 27064 (PRJNA19249) is marked with a red line

The 138 kb region is inserted at position 6,298,508 of the chromosome relative to the PRJNA19249, which is at present the most complete public genome sequence of *S*. *clavuligerus* ATCC 27064 (Fig. 2). Upon investigation of the insertion joint, no inverted repeats, palindromes, or other elements that could indicate involvement of an integrase or transposase could be found. Instead, a moderately conserved direct repeat of 19 bp is positioned at the borders of the 138 kb region (Fig. 2). This may facilitate excision of the 138 kb region by homologous recombination, perhaps as a repair after a double-strand break or other recombinase-assisted event. Such event would indeed resolve into the situation found in the published ATCC genomes, having one of the direct repeats conserved at the excision site (Fig. 2).

#### Early *S. clavuligerus* ATCC 27064 cultures produce teleocidins

In agreement with Li et al. (2018), the analysis of the 138 kb genomic region (position 441,384–579,552 of PRJNA360257) with AntiSMASH 3.0 predicted the presence of several secondary metabolite gene clusters, and among them, putative teleocidin biosynthetic genes. The genetic organization of this cluster (Fig. 1) is identical to the recently reported situation in *Streptomyces blastmyceticus* (Abe 2018); however, the gene context into which the teleocidin cluster is placed is completely different (Supplemental Fig. S7). The identity scores compared to *S*. *blastmyceticus* TleA, TleB, and TleC are 68%, 84%, and 66%, respectively. The *tleA* and *tleB* genes are interspaced with a putative MbtH-encoding gene (BV309_02880). Interestingly, in analogy with the *S*. *blastmyceticus*, a methyltransferase gene *tleD*, which is needed for conversion of teleocidin A to teleocidin B (Abe 2018), is not associated with the cluster, nor can be found within the 138 kb DNA region. Instead, a putative TleD homologue with 66% identity is encoded by a SAM-dependent methyltransferase gene (BV309_32160), conserved in the giant linear plasmid pSCL4 of all published genomes of *S*. *clavuligerus* strains.

**Fig. 3:**
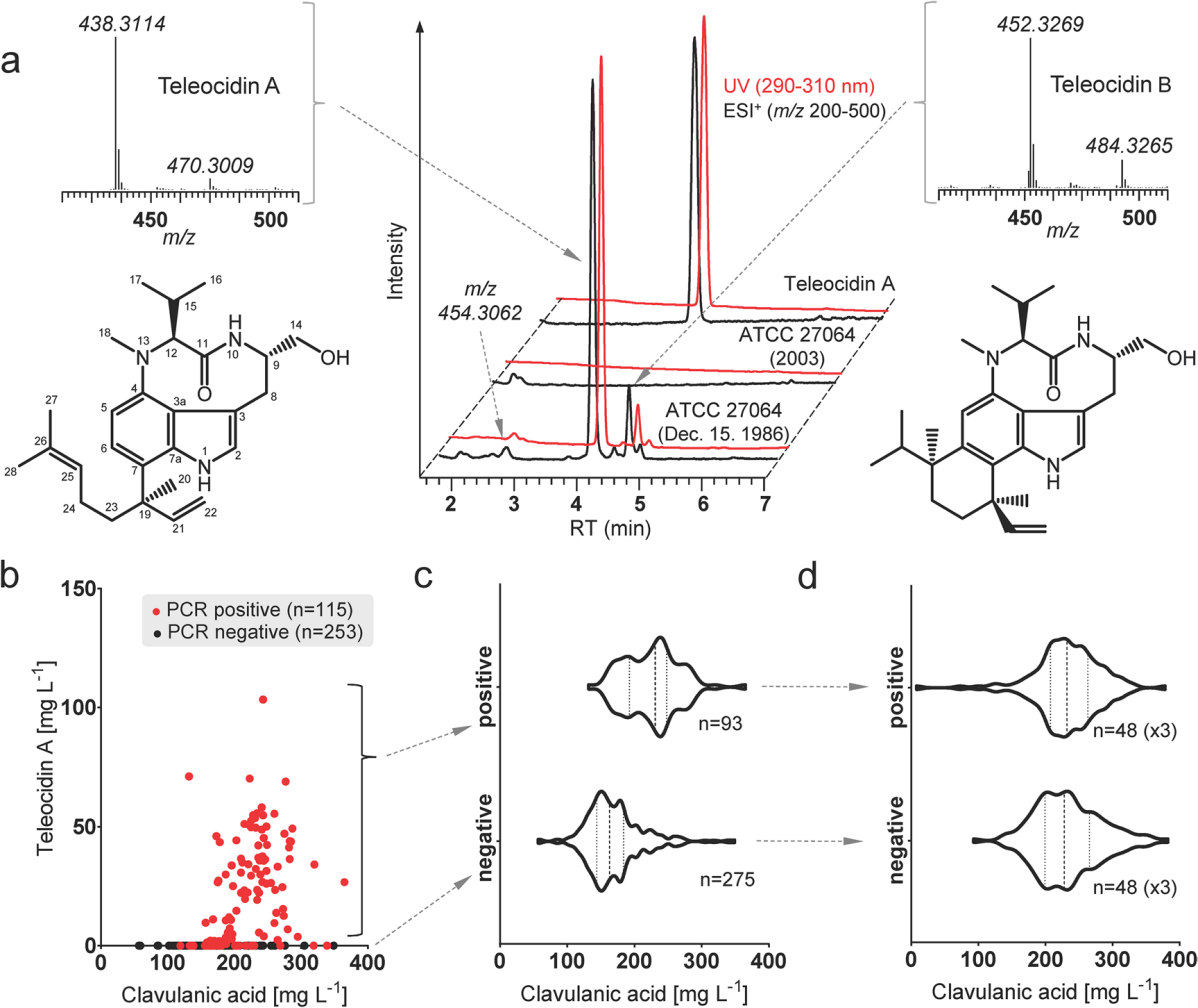
**a** LC–MS and UV chromatograms of *S*. *clavuligerus* ATCC 27064 “Dec. 15. 1986” cultures vs. more recent *S*. *clavuligerus* ATCC 27064 stock (obtained in 2003) after 5 days of cultivation. **b** Prevalence of teleocidin-producing subpopulation within the original *S*. *clavuligerus* ATCC 27064 “Dec. 15. 1986” culture. Randomly picked colonies (*n* = 384) were grown in deep well plates for 5 days. Wells with obvious lack of growth (*n* = 16) have been excluded. Red dots represent colonies with positive colony-PCR result using primer pair 6. Out of 115 PCR-positive colonies, 22 did not produce teleocidins; none of the PCR-negative colonies produced teleodicins. **c** CA titers of teleocidin-producing vs. teleocidin-nonproducing subpopulation. Production of CA was higher in the teleocidin-producing colonies (Δ median: 68.0, Mann–Whitney test: *P* < 0.0001), likely a bias due to differences in growth dynamics within the population (Supplemental Fig. S12). **d** CA titers upon re-cultivation of top 48 CA producers from each the teleocidin-producing and teleocidin-nonproducing subpopulation after 4 days of cultivation in a 96 deep well plate; no significant difference in CA productivity was found (Δ mean: 0.271, repeated measures two-way ANOVA: *P* = 0.9586). Detailed statistical analysis is provided in Supplemental Table S4

LC–MS analysis of liquid cultures of *S*. *clavuligerus* ATCC 27064 from the “1986” vial revealed the presence of teleocidin A as the major, and teleocidin B as the minor indolactam compound (Fig. 3a). Conversely, none were detected in cultures of later ATCC vials (Fig. 3a). The indolactams were found mostly associated with the mycelium of the *S*. *clavuligerus* cultures and only traces could be detected in culture supernatants. HRMS data for Teleocidin A (C_27_H_40_N_3_O_2_; calc.: 438.3115; found: 438.3114; Δ = 0.2 ppm) and Teleocidin B (C_28_H_42_N_3_O_2_; calc.: 452.3272; found: 452.3269; Δ = 0.7 ppm) as well as UV-absorption spectra and chromatographic behavior (Fig. 3a) of these compounds are in agreement with authentic samples and the published data (Hitotsuyanagi et al. 1984; Takashima et al. 1962). In addition, we have observed the presence of compounds with m/z [M + H^+^] = 454 and characteristic UV absorption spectra that could indicate teleocidin-related compound. HRMS data suggest molecular formula C_27_H_39_N_3_O_3_ (calculated: 454.3064; found: 454.3062; Δ = 0.4 ppm). This formula corresponds, among others, to 2-oxo-teleocidin A (JBIR-31), a teleocidin analog found in *Streptomyces* sp. NBRC 105896 (Izumikawa et al. 2009). 2-Oxo congeners of indolactam V, the intermediate in the teleocidin synthesis, have also been found to accumulate in other *Streptomyces* upon heterologous expression of the pathway (Zhang et al. 2016).

Whole-broth extracts were used to isolate and confirm the identity of the major compound. Subsequent HPLC-prep purification gave ~ 38 mg of teleocidin (lyngbyatoxin) A in form of off-white to brownish amorphous powder. The ^1^H-NMR, ^13^C-NMR, and HRMS spectra were found in agreement with the published data (Supplemental Fig. S8-S10; Supplemental Table S2-S3; Cardellina et al. 1979; Takashima et al. 1962), and indicated presence of two diastereoisomers at position 19 in the ratio ~ 6:1. The major diastereoisomer was determined to be teleocidin A1 and the minor teleocidin A2.

#### Population diversity of early *S. clavuligerus* ATCC 27064 cultures

MTP testing of 368 randomly picked single colonies from the plated culture of *S*. *clavuligerus* ATCC 27064 (the 1986 vial) has shown that only a part of the population (*n* = 93) produces teleocidins (Fig. 3b). In parallel, we have performed analytical PCR (primer pair 6) for the presence of the teleocidin genes in each of the tested colonies. A third of the population (*n* = 115) was PCR-positive and all of the teleocidin-producing colonies were found within this group (Fig. 3b). Some morphological differences were observed between the colonies on the primary plating (Supplemental Fig. S11), raising hope for a visual distinction between the genotypes and a plausible explanation for population enrichment toward the contemporary ATCC strain. Unfortunately, we could not find any correlation between morphology and ability to produce teleocidins. In addition, these differences were no longer visible after sub-plating (Supplemental Fig. S11). The initial population screening showed a positive correlation between productivity of CA and production of teleocidins (Fig. 3c); however, we attribute this correlation to bias from growth dynamics differences between the colonies in the assay. Oxygen demand dynamics of the two subpopulations, measured under constant conditions (Supplemental Fig. S12), suggest that respiration rates in the exponential phase are higher for the teleocidin-negative population. Due to earlier nutrient depletion, the faster growing cultures typically show arrest of CA production curve (Supplementary Fig. S13, S14). To address this issue, we subsequently subcultured and tested 48 of the top CA producers from each the teleocidin-positive pool and the teleocidin-negative pool in triplicates. By providing a smaller, more consistent inoculum across the tested population and by sampling on the 4^th^ day of the cultivation, the above effects have been avoided. The results show no statistically relevant differences in CA productivity between the two subpopulations (Fig. 3d).

Monoisolates of entirely teleocidin-positive populations (e.g., K4567) were obtained from the mixed population after the second sub-plating cycle. Teleocidin-negative monoisolates, on the other hand, were homogenous already at the first sub-plating round. The monoisolates showed stable genotypes and phenotypes for several successive sub-plating rounds and throughout the genetic modification procedures.

**Fig. 4:**
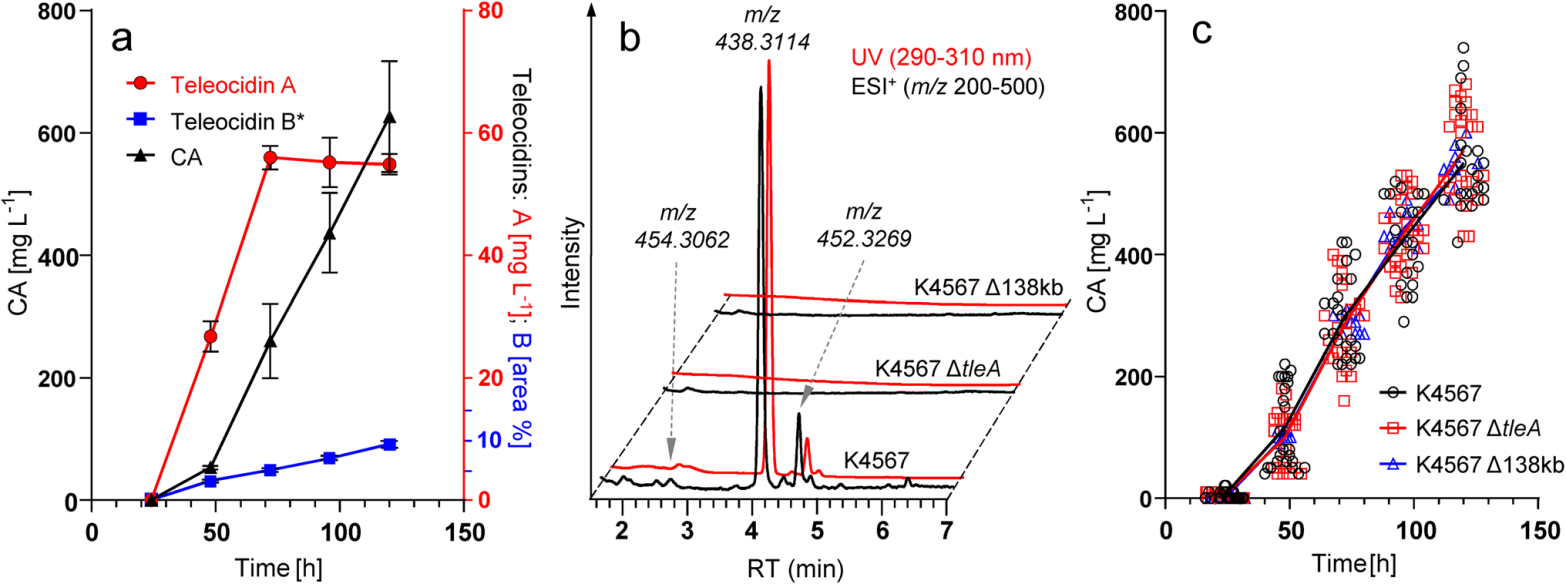
**a** Accumulation of CA and teleocidins in cultures of *S*. *clavuligerus* K4567. Data are means and SD of three independent cultivations. Teleodicin A concentrations are plotted to the secondary axis. *Teleocidin B is given as UV peak area percent (290–310 nm) on the secondary axis. **b** LC–MS analysis of teleocidin production in *S*. *clavuligerus* K4567 and the deletion mutants. **c** Comparison of CA productivity for *S*. *clavuligerus* K4567 (*n* = 21), K4567 Δ*tleA* (*n* = 24), and K4567 Δ138 kb (*n* = 9), compiled from multiple shake-flask experiments. The lines are connecting means of values at each sampling point (24, 48, 72, 96, and 120 h). Statistical analysis was performed using one-way ANOVA with Tukey’s multiple comparison for each time point separately (Supplemental Table S5)

The teleocidin-positive monoisolate (*S*. *clavuligerus* K4567) was then cultivated in shake flasks and cultures analyzed periodically for production of CA and teleocidins, as well as nutrient and oxygen consumption (Fig. 4a; Supplemental Fig. S13, S14). The production of teleocidin A reaches a plateau already at 72 h of the process, typically peaking and remaining at ~ 55 mg L^−1^. In contrast, the concentration of CA is increasing throughout the process, reaching ~ 550 mg L^−1^ after 120 h. Interestingly, teleocidin B is also being formed continuously, arriving to the final ~ 10% of teleocidin A (UV peak area percent) at the end of the cultivation (Fig. 4a).

#### Deletion of *S. clavuligerustleA* results in loss of teleocidin production

A mutant with deletion of the *tleA* gene was isolated using double crossover, positive–negative selection marker approach (Supplemental Fig. S1). The deletion was confirmed by PCR (Supplemental Fig. S2, S3). Comparison of the shake flask cultures of *∆tleA* strain with the w.t. teleocidin-producing strain (K4567) revealed complete absence of all teleocidin production in the mutant strain, namely the teleocidin A, and all putative congeners (Fig. 4b). The same results were obtained when the complete 138 kb region was deleted (Fig. 4b). As with the native teleocidin-negative subpopulation, faster growth dynamics were observed with the K4567 *∆*138 kb strain. This was easily mitigated by adjusting the inoculum size for the seed cultures (Supplemental Fig. S14). Subsequent experiments with the deletion strains, regardless of the length of the deleted region, showed no statistically significant differences in productivity of clavulanic acid (Fig. 4c, Supplemental Table S5).

## Discussion

Genome plasticity with large genomic rearrangements is frequently observed with *Streptomyces* (Hoff et al. 2018); therefore, population diversification with *S*. *clavuligerus* ATCC 27064 is not surprising. Due to its capability to produce CA, this organism has been disseminated from a single initial source to laboratories and industrial facilities all over the world. We show in our work that the early stocks of *S*. *clavuligerus* ATCC 27064 contained another genotype, which has been lost from the subsequent collection stocks, and has remained obscure to the scientific community. Most importantly, this genotype harbors teleocidin biosynthetic genes, which are located on a distinct 138 kb chromosomal region and support accumulation of significant amounts of these highly toxic compounds in cultures of *S. clavuligerus*.

Nevertheless, it appears that this genotype was not entirely lost. A recent study identified the presence of indolactam V, a teleocidin intermediate, in extracts of one of the *S*. *clavuligerus* ATCC 27064 lines (AbuSara et al. 2019); an observation that cannot be explained with genomic data published thus far. In a sense, such examples invalidate thus far frequently uncontested assumptions that strains disseminated from type strain collections and maintained independently at various laboratories can be regarded equal. Our work provides a genome sequence for the teleocidin-producing genotype of *S*. *clavuligerus* ATCC 27064 and a corresponding monoisolate to be available again to the scientific community, allowing reinterpretation and perhaps offering new meaning to unexplained observations.

In addition, at least one of the industrial *S*. *clavuligerus* strains for commercial production of CA originating from ATCC 27064, the *S*. *clavuligerus* F613-1, holds the genetic potential for production of teleocidins (Cao et al. 2016; Li et al. 2018). Interestingly, in contrast to contemporary ATCC strains, this industrial producer retained the 138 kb region despite undergoing vast genomic rearrangements, which resulted in more than 1 Mb genome reduction (Li et al. 2018). One plausible explanation is that a genetic element within the 138 kb region is complementing for a paralogous function lost through the genome reduction, thereby causing a pressure for preservation of the 138 kb region. Admittedly, not knowing the selection procedures and lab workflows used in development and maintenance of the F613-1 strain, the latter is highly speculative, and other effects (e.g., differences in growth dynamics) may play an important role in conservation of the 138 kb region.

Evidence for teleocidin genes in industrial strains and the sheer scale of CA production globally, raises questions about safety and environmental impact, which need to be addressed appropriately. Teleocidins are water-insoluble and unstable in acidic environment (Takashima et al. 1962). This is fortunate because the established industrial processes for production of CA use three key steps (Saudagar et al. 2008; Danielsson et al. 2006), which effectively prevent contamination of CA with teleocidins. (i) After fermentation, the mycelium is most often separated from the aqueous CA solution by means of filtration or centrifugation, leading teleocidins, which are associated with mycelium phase to the waste stream. (ii) The aqueous CA solution is then acidified to pH ~ 2, to allow extraction of CA to water-immiscible organic solvent. This acidification would degrade any teleocidin carry-over from the filtration step. (iii) CA is precipitated from organic solvent by formation of salt with addition of organic or inorganic base, rendering CA insoluble in organic solvents. Again, teleocidins cannot be carried over through this purification stage. The risk of teleocidin carry-over to the final CA product is therefore minor.

On the other hand, there is a strong concern about the environmental impact coming from the waste mycelium streams, potentially contaminated with teleocidin. In fact, there has already been a report where workers in industrial fermentation process with another *Streptomyces* species complained about skin irritation, which was subsequently attributed to teleocidins (Sugimura 1986). These toxins, which are also produced by a filamentous blue-green alga *Moorea producens* (formerly *Lyngbya majuscula*), are known to cause acute dermatitis called “swimmer’s itch” (Cardellina et al. 1979; Osborne et al. 2001). Occasional acute poisonings by ingestion of marine turtle meat (*Chelonia mydas*) have been reported in Indo-Pacific, several with fatal outcomes (Champetier et al. 1998; Halstead 1970). At least in one case, the intoxication was linked to the accumulation of algal teleocidin A (lyngbyatoxin A) in the food chain (Yasumoto 1998).

Little is known about the effects of long-term exposure of humans to subtoxic doses of teleocidins; however, given their tumor-promoting activity (Fujiki et al. 1981; Osborne et al. 2001) and toxicity (Ito et al. 2002), caution is appropriate. With improper waste mycelium management strategy and more critically with potential reuse of the waste mycelium biomass (e.g., as fertilizer, animal feedstock etc.), teleocidins may enter human or animal diet. Therefore, a risk assessment should be made for each specific situation where large amounts of *S*. *clavuligerus* biomass are being generated. Importantly, our results show that deletion of *tle* genes does not affect CA productivity potential in *S*. *clavuligerus* ATCC 27064 genomic background, pointing out to a potential solution for the undesired impacts of teleocidin co-production also with industrial *S*. *clavuligerus* strains.

## Supplementary Information

Below is the link to the electronic supplementary material.

Supplementary file1 (PDF 3369 KB)

## Data Availability

The strain *S*. *clavuligerus* K4567 has been deposited at DSMZ under DSM33546, other strains and plasmids used in this study are available upon request. The genome data for *S*. *clavuligerus* K4567 is available under NCBI BioProject PRJNA360257. All other data generated or analyzed during this study are included in this published article and its supplementary information files.
