# Supplementary material for: Teleocidin-producing genotype of Streptomyces clavuligerus ATCC 27064
Source: Appl Microbiol Biotechnol. 2022 Feb 9;106(4):1521–30. doi: 10.1007/s00253-022-11805-5 (PMC8882083; doi:10.1007/s00253-022-11805-5)
Supplement: Supplementary file 1 — Supplementary file1 (PDF 3369 KB) [file 253_2022_11805_MOESM1_ESM.pdf]

Applied Microbiology and Biotechnology

Supplementary Information for

Teleocidin-producing genotype of *Streptomyces clavuligerus* ATCC 27064.

*Petra Pivk Lukančič, Tjaša Drčar, Robert Bruccoleri, Martin Črnugelj and Peter Mrak**

*peter.mrak@novartis.com

This PDF file includes:

- Figs. S1 to S14
- Tables S1 to S5

Table S1. PCR primers used in this work. Primer pairs 1 to 4 were used for cloning of the editing template fragments. Restriction sites introduced for cloning are underlined. Primer pairs 5-8 were used for genotype screening in the native population and for confirmation of the deletion mutant genotypes. Due to the length of the deletions in the mutants, two distinct forward primers were used; one pair positioned externally of the deletion locus and providing PCR product only in case of mutant genotype (5 & 7) and the second pair positioned within the deletion locus, yielding PCR product only in case of the w.t. genotype (6 & 8). DNA templates were prepared from a single colony. The colony was picked from agar plate and homogenized with a plastic pestle in 200 µL Tris-EDTA buffer (TE), pH 8.0. Sample was then frozen at -80°C for at least 5 min, heated up to 80°C, incubated for 5 min and left to cool. In case of liquid cultures, 10 µL of broth was filtered, resuspended in 100 µL TE and thermally treated as above. The PCR reaction was carried out with Q5 High-Fidelity DNA Polymerase (New England Biolabs). 25 µL reaction mixture was composed of: Q5 Reaction Buffer (1x), 200 µM dNTP, 0,5 µM of each primer, Q5 High GC Enhancer (1x), 2 µL of the template and 0.5 unit of the enzyme. The thermal profile started with denaturation step at 98 °C for 5 min. The thermal cycle for the following 35 cycles was 95 °C for 30 sec (denaturation step), 62 °C for 20 sec (annealing step), and 72 °C for 90 seconds (extension step). The final elongation step was carried out at 72 °C for 7 min.

#	Primer pair	Amplicon length	Amplicon position
1	GCGCGGATCCTTCCCGTGCGAGCGCGTGAC<br>GCGCCAATTGGAACCCCCCGCGCGC	1436	560216-561651
2	GCGCCAATTGATCCTCGGCGACCCCCG<br>GCGCAAGCTTCGCGGCGACGATACGCCG	1450	549581-551030
3	GCGCGGATCCGTGATGCGGCACGGACTCG<br>GCGCCAATTGGCTGGTGTTCTCCTTGGTGATCG	1803	573797-575599
4	GCGCCAATTGCGAGGTCCTCTTCGCCACCG<br>GCGCAAGCTTGCCGTGCCTGTCCGCAAG	1846	442186-444031
5	TAGGAGACGAAGTTCTGGGTCAGGGA<br>AGCCTGCCGATGTAGTCGTAGAGCA	1273	*550615-561066
6	CTTCGACTCGGTCCTGGACCATCT<br>AGCCTGCCGATGTAGTCGTAGAGCA	1337	550615-551951
7	ACCTCTACAACAAGCCGATGAACCAGA<br>GCTGATCGTCGGATACGAGGGCA	978	*443434-574170
8	GAGTCGGAACACCACGTCCTGACC<br>GCTGATCGTCGGATACGAGGGCA	1005	443434-444438

*Theoretical amplicon position in reference to the K4567 w.t. genome.

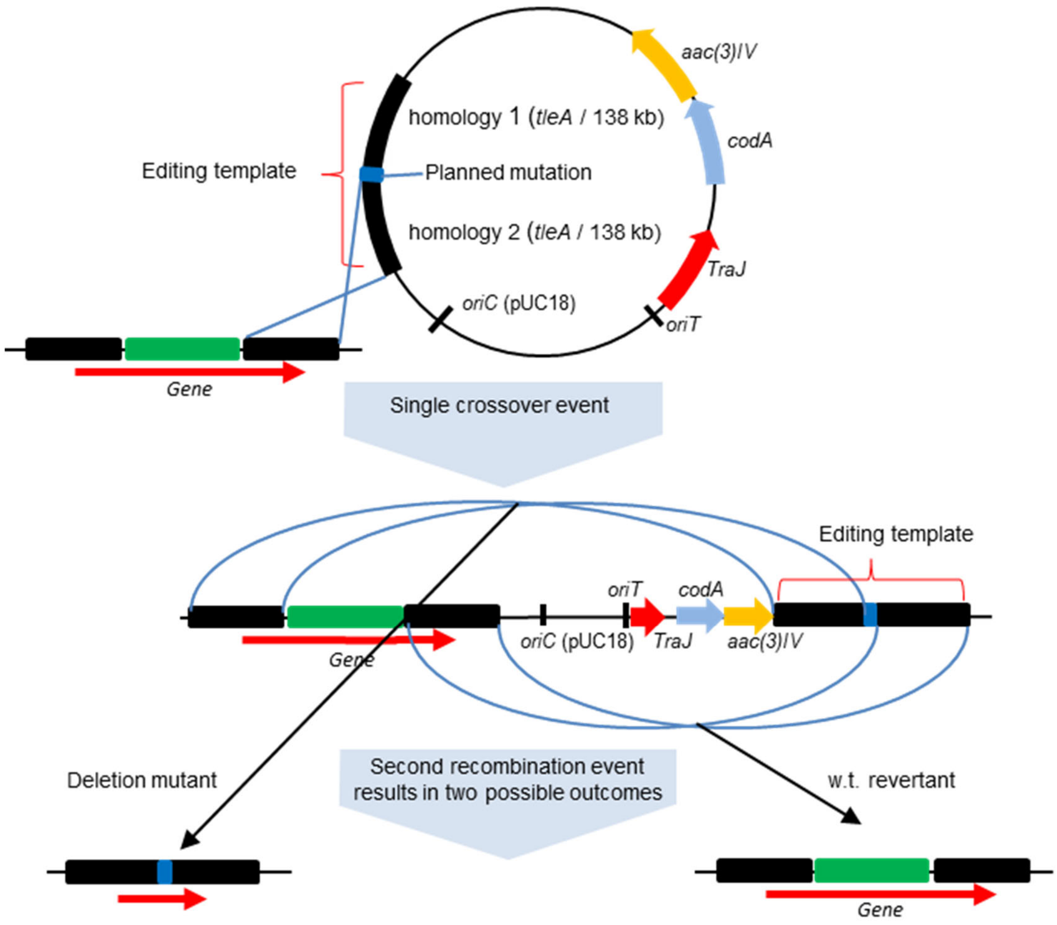

Fig. S1. Schematic representation of the vectors used for introduction of deletions into the *S. clavuligerus* genome.

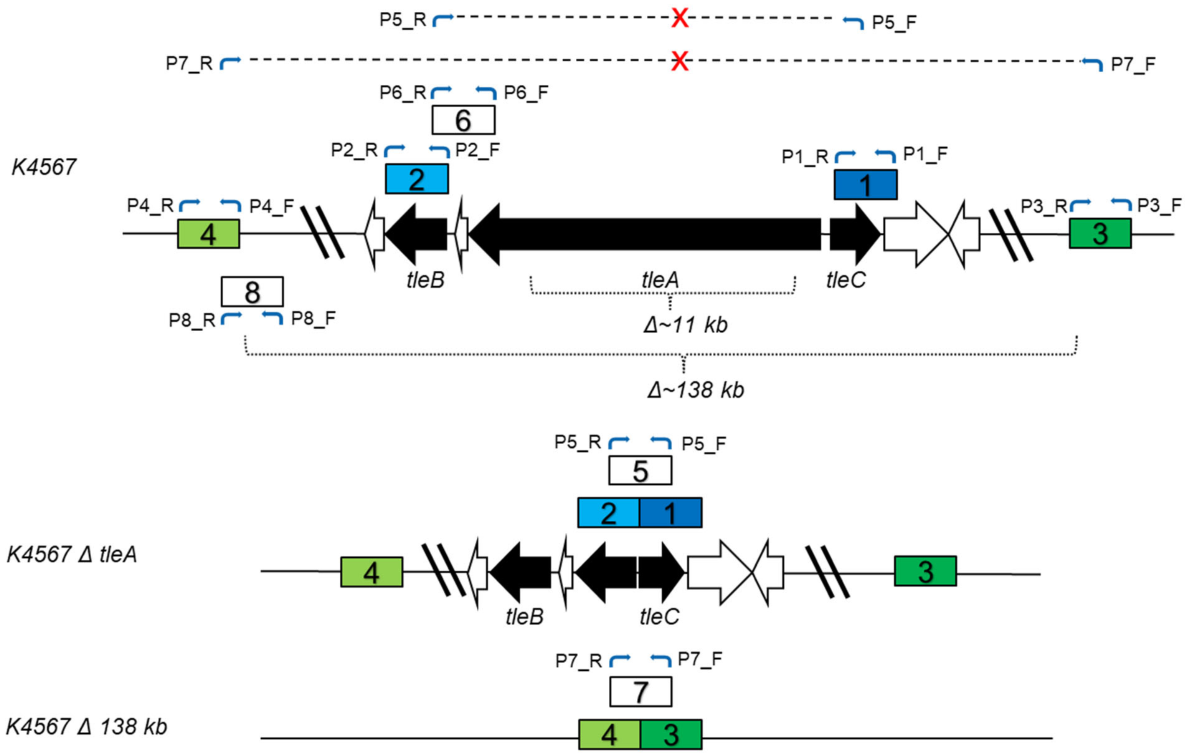

Fig. S2. Schematic representation of the PCR amplicons (boxes) and primers (blue arrows) used for verification of deletions introduced to *S. clavuligerus* K4567 genome. Genetic situation in K4567, K4567 Δ*tleA* and K4567 Δ138kb are shown. Primer labels consist of primer pair number (Table S1) and F (forward) / R (reverse) denominations. The numbered boxes represent amplicons according to table S1. Primer pairs 1 through 4 (colored boxes) were used to amplify targeting regions for homologous recombination. Primer pairs 5 through 8 (empty boxes) were used for genotype verification. Due to oversize length of the amplicon, primer pairs 5 and 7 do not form PCR product with K4567 genome template.

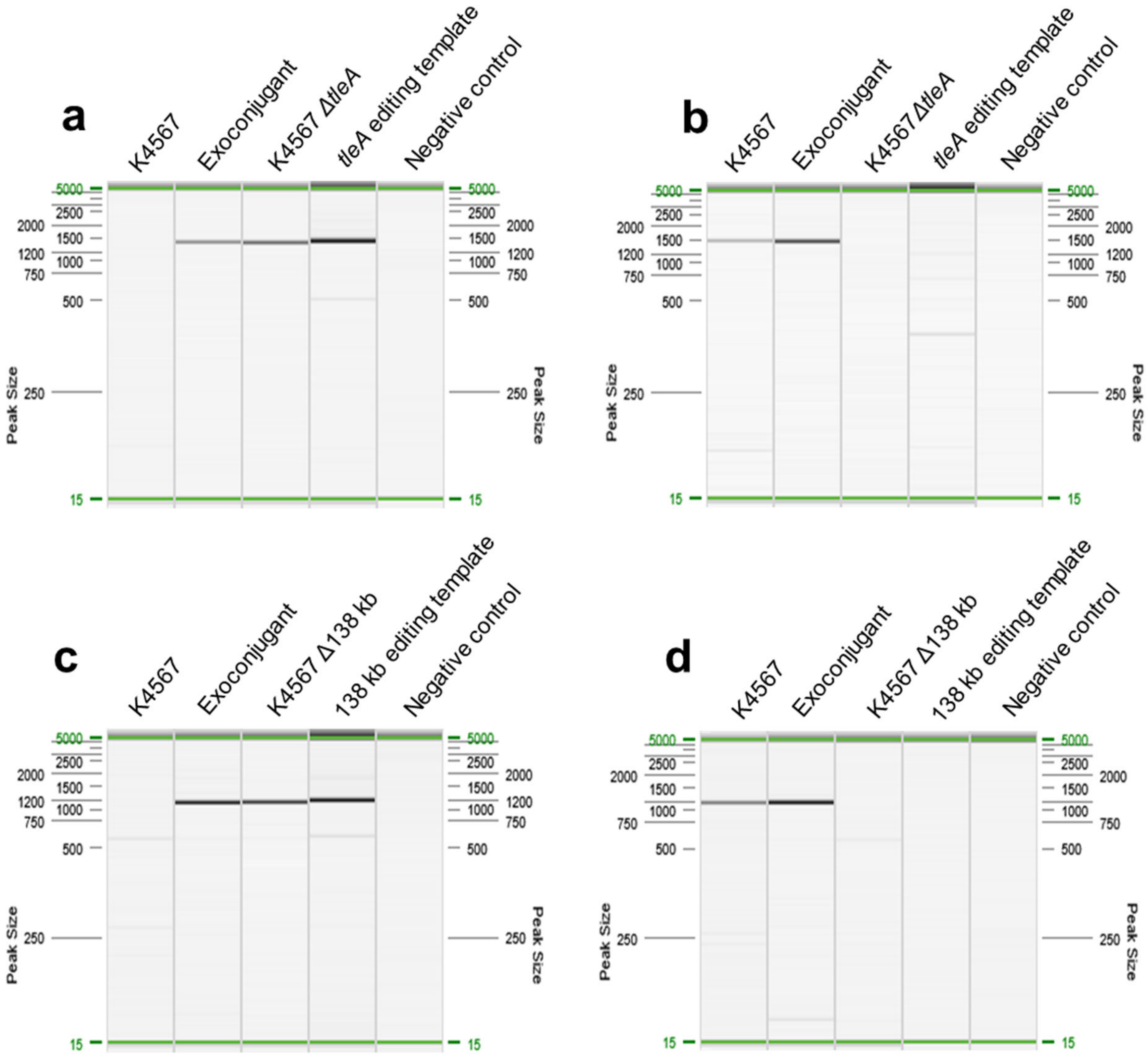

Fig. S3. Electropherograms of genotype confirmation PCRs. Capillary gel electrophoresis was used to analyze the PCR reactions. The unit of scale is [bp]. **a** Confirmation of K4567 Δ*tleA* genotype, primer set 5; **b** Confirmation of K4567 Δ*tleA* genotype, primer set 6. **c** Confirmation of K4567 Δ138kb genotype, primer set 7. **d** Confirmation of K4567 Δ138kb genotype primer set 8.

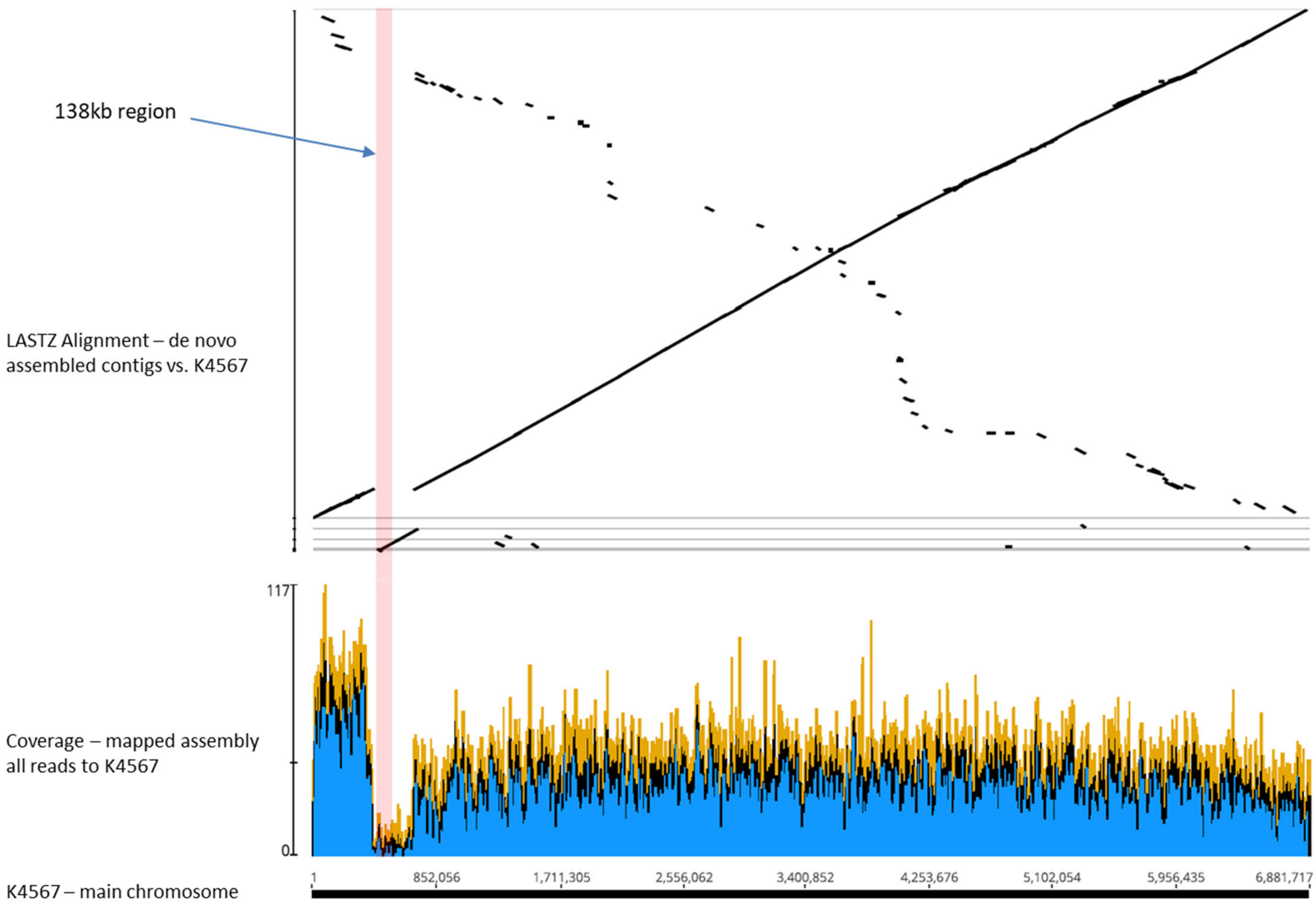

Fig. S4. Preliminary sequencing data of the mixed population *S. clavuligerus* ATCC 27064 culture from the earliest ATCC vial (labeled "Dec. 15. 1986" by the ATCC). *De novo* assembled contigs, aligned with the main chromosome of *S. clavuligerus* K4567 are shown at the top (LASTZ genome alignment). One of the contigs is homologous to the mysterious 138kb region. Coverage of all reads mapped to the *S. clavuligerus* K4567 main chromosome is shown at the bottom of the figure. Several genome variants were predicted with main variation at the locus 0 to ~ 680.000. Subsequent sequencing data for one of these variants, the monoisolate K4567, are described in the main text.

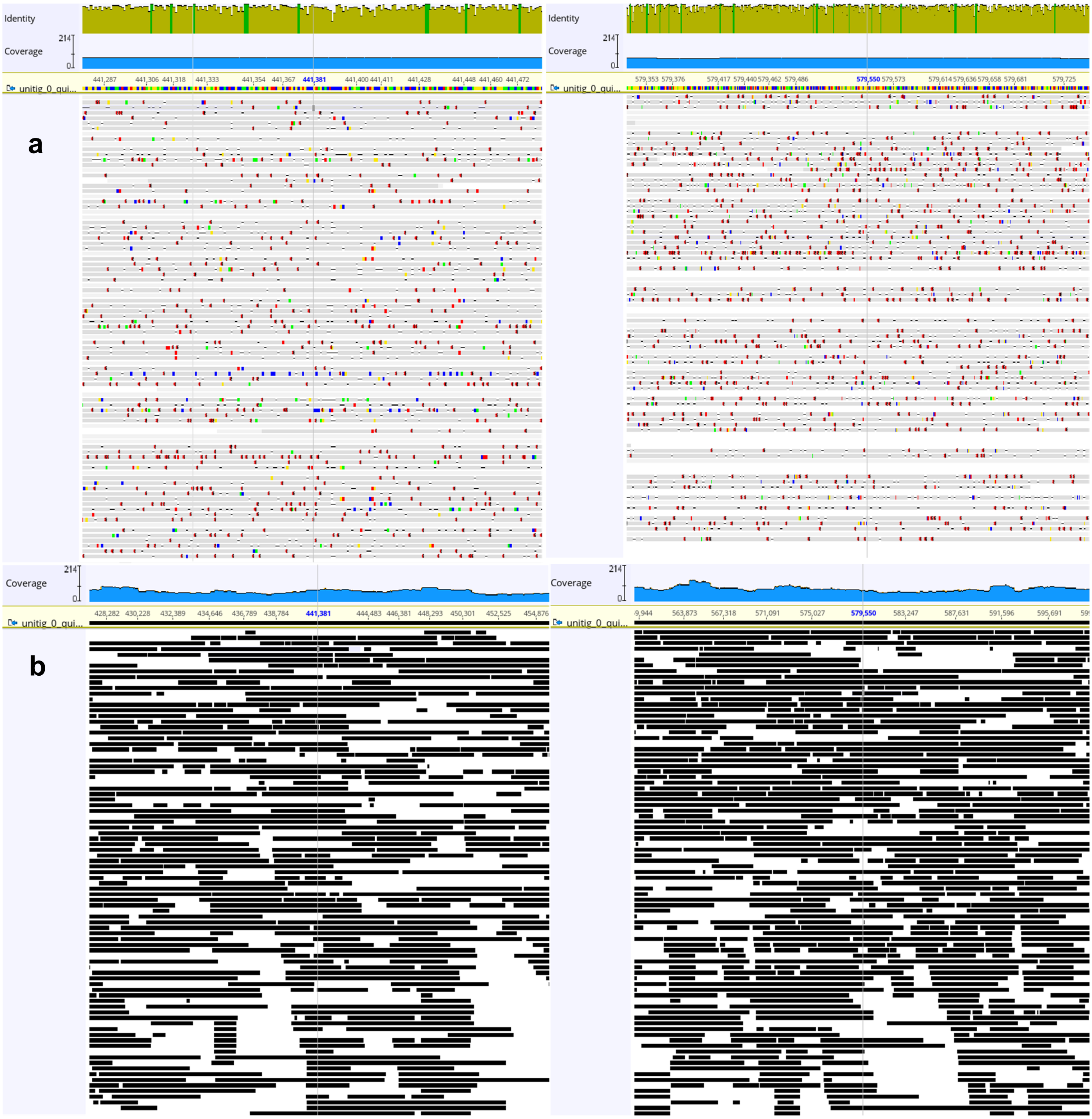

Fig. S5. The alignments that demonstrate the correct assembly of the junctions on the 138kb region borders (position 441.381-579.550 of K4567 main chromosome) were obtained by converting the PacBio HDF files from the sequencing movies into BAM file format (bax2bam function in PacBio's SMRTLink 3.0.0). The alignments were performed using the pbmm2 program from PacBio's SMRTLink 10.0.0 with all the subreads contained in the merged BAM files and using the assembled K4567 genome as the reference. The alignments were visualized using the Geneious Prime software. **a** Insertion bases in raw reads not included in consensus (>10% abundance) are shown as red markers. Mismatches are shown as colored boxes (T=green, A=red, G=yellow, C=blue). Identity score for each column is shown at the top. **b** Zoom-out view of the 138kb region joint showing alignment of uninterrupted raw reads onto the assembled genome reference.

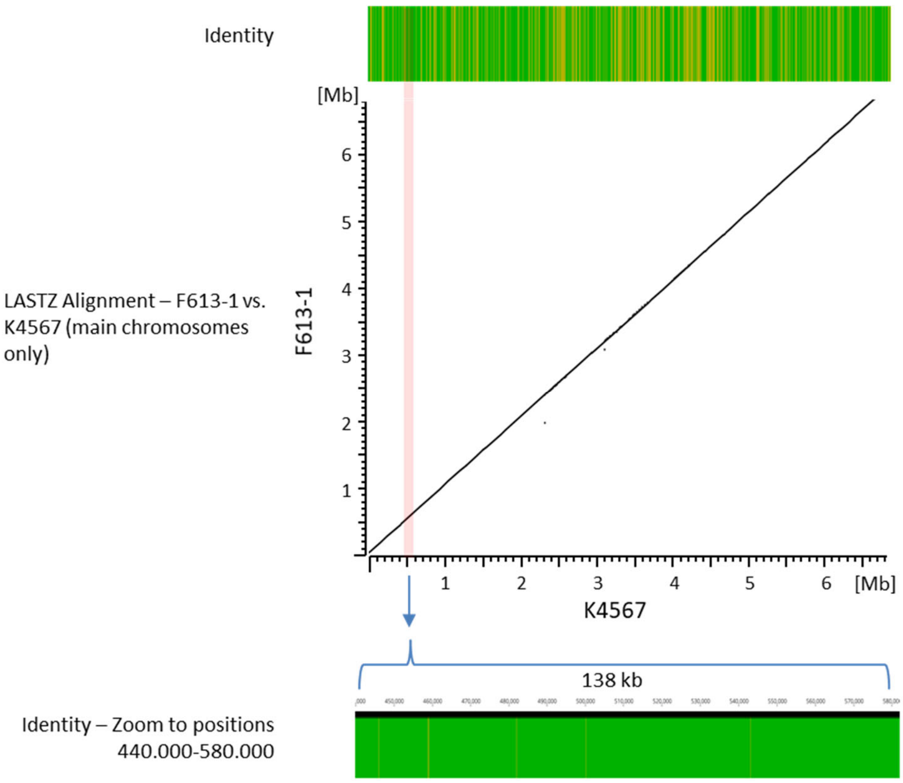

Fig. S6. LASTZ alignment of main chromosomes of *S. clavuligerus* strains K4567 (PRJNA360257) vs. *S. clavuligerus* F613-1 (PRJNA329150). Identity score for the whole alignment is shown at the top (green = identity, yellow = mismatches at single nucleotide level). A zoom to the 138kb region (position 441.381-579.550 of K4567 main chromosome) with identity score is shown at the bottom.

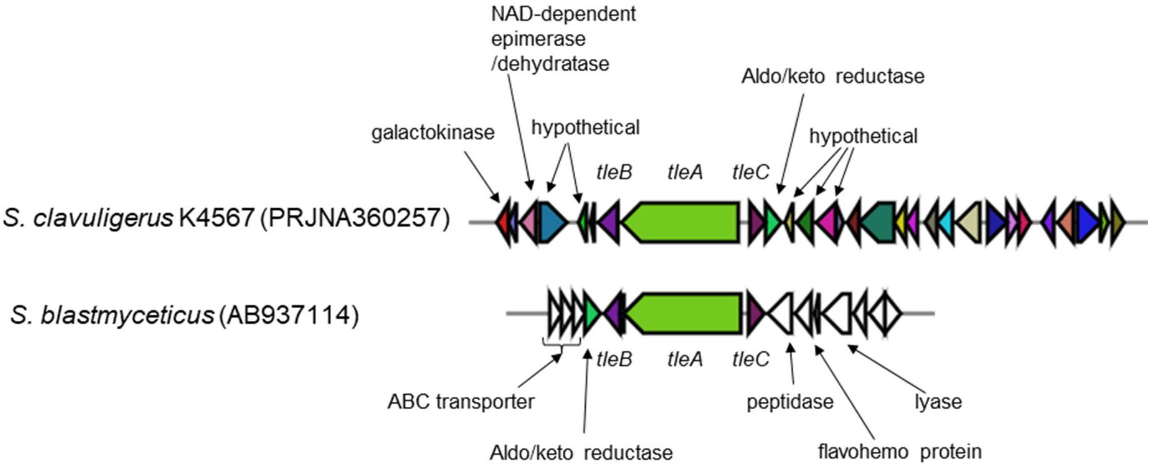

Fig. S7. Comparison of gene topology surrounding the teleocidin gene cluster in *S. clavuligerus* K4567 (PRJNA360257) vs. *S. blastmyceticus* (AB937114). Despite being the closest relatives sequence-vise, the differences in immediate gene topology around the clusters indicate significant evolutionary distance.

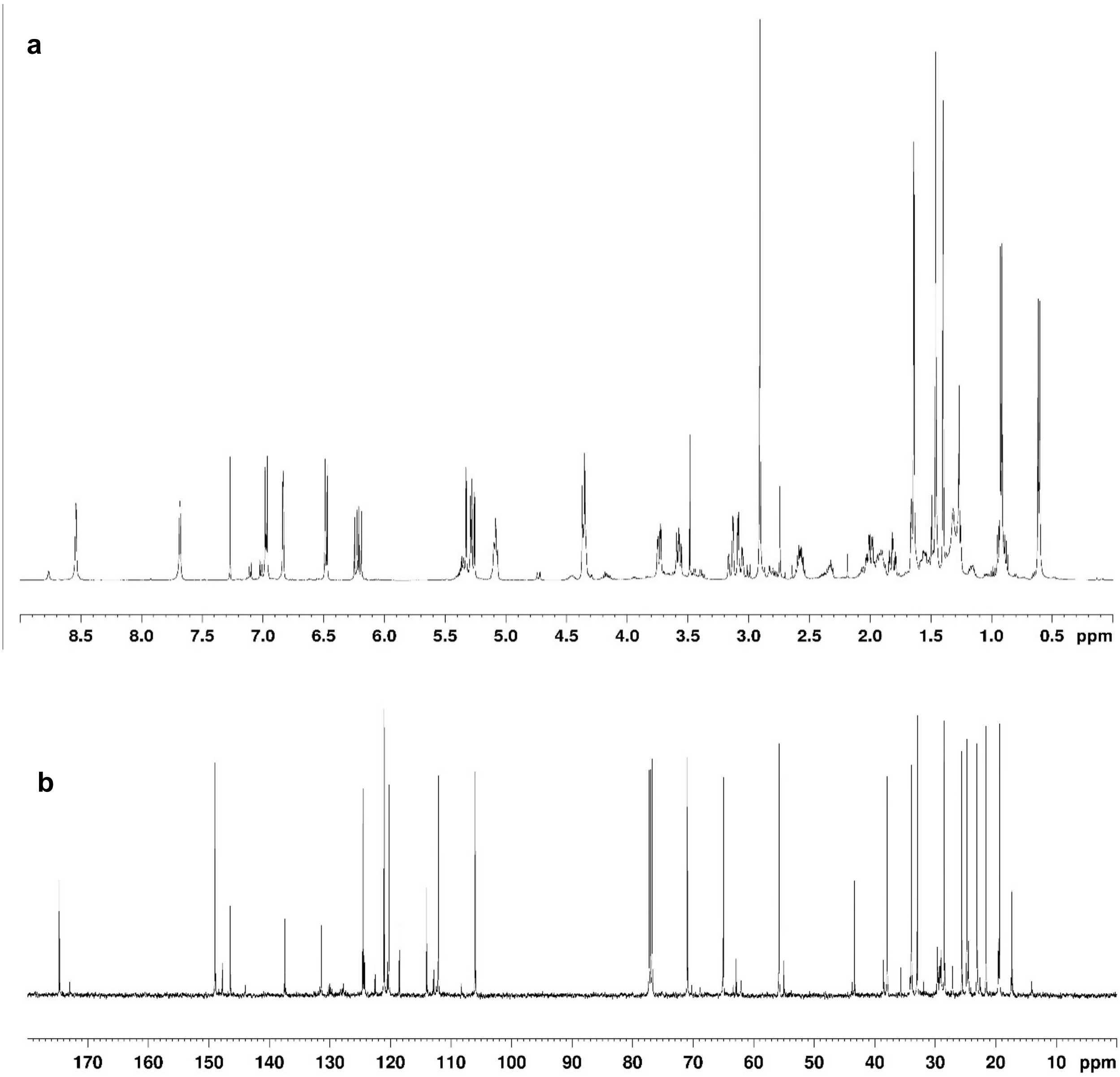

Fig. S8. Raw NMR spectra supporting the structure determination of teleocidin A. **a** ^{1}H-NMR (500 MHz, $CDCl_3$) spectrum is shown. **b** ^{13}C-NMR (150 MHz, $CDCl_3$) spectrum is shown.

a

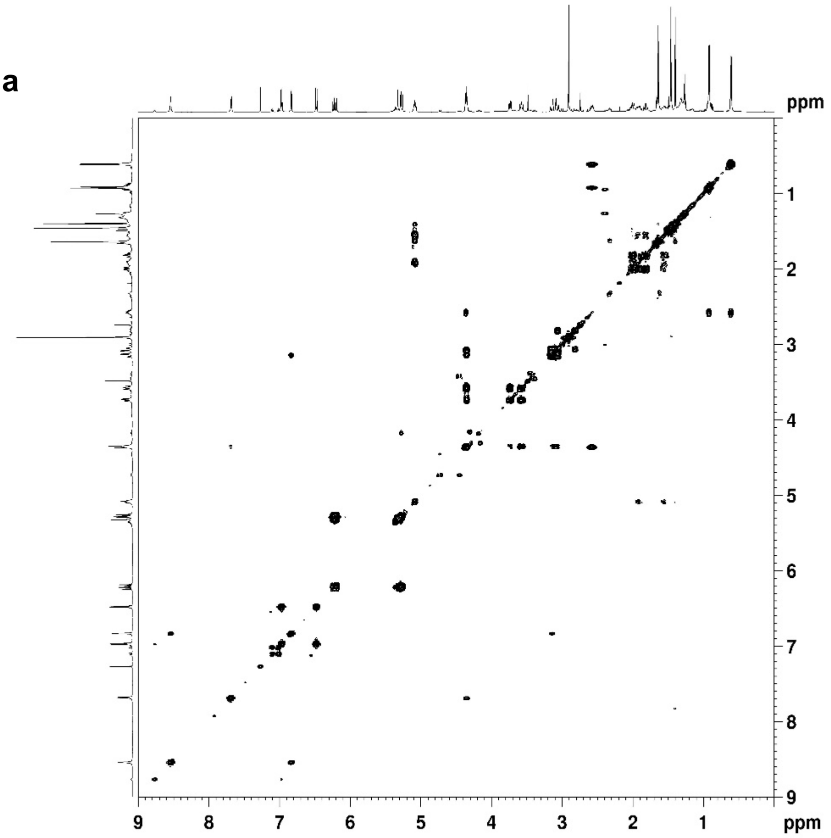

b

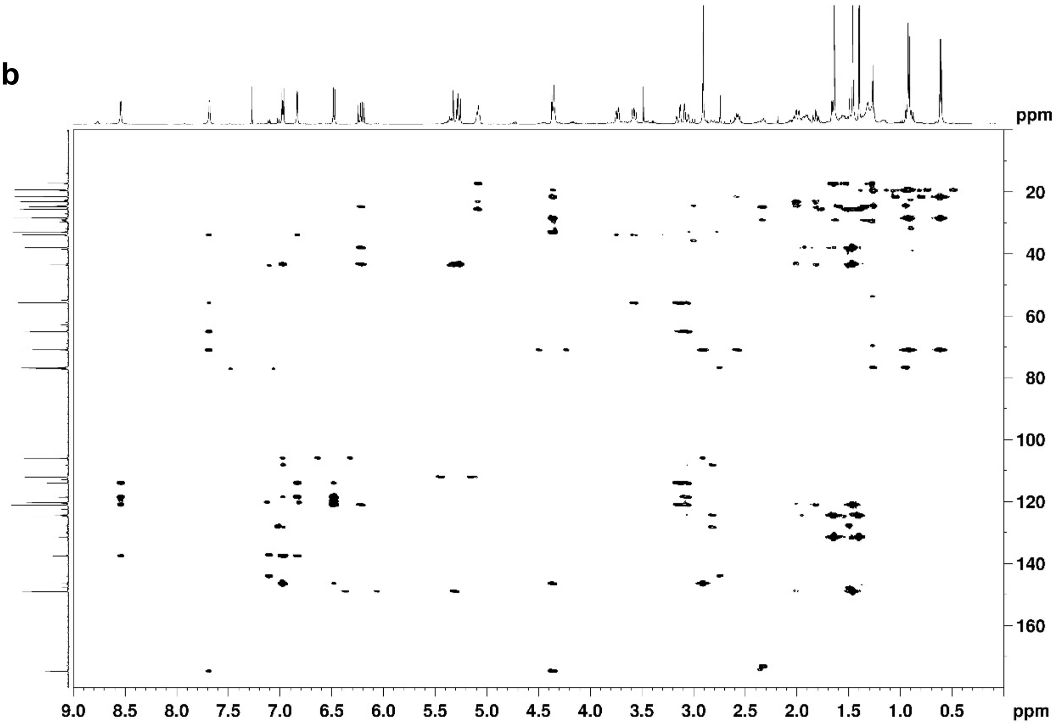

Fig. S9. a COSY ^{1}H-NMR spectrum correlation for teleocidin A. **b** HMBC NMR spectrum correlation for teleocidin A.

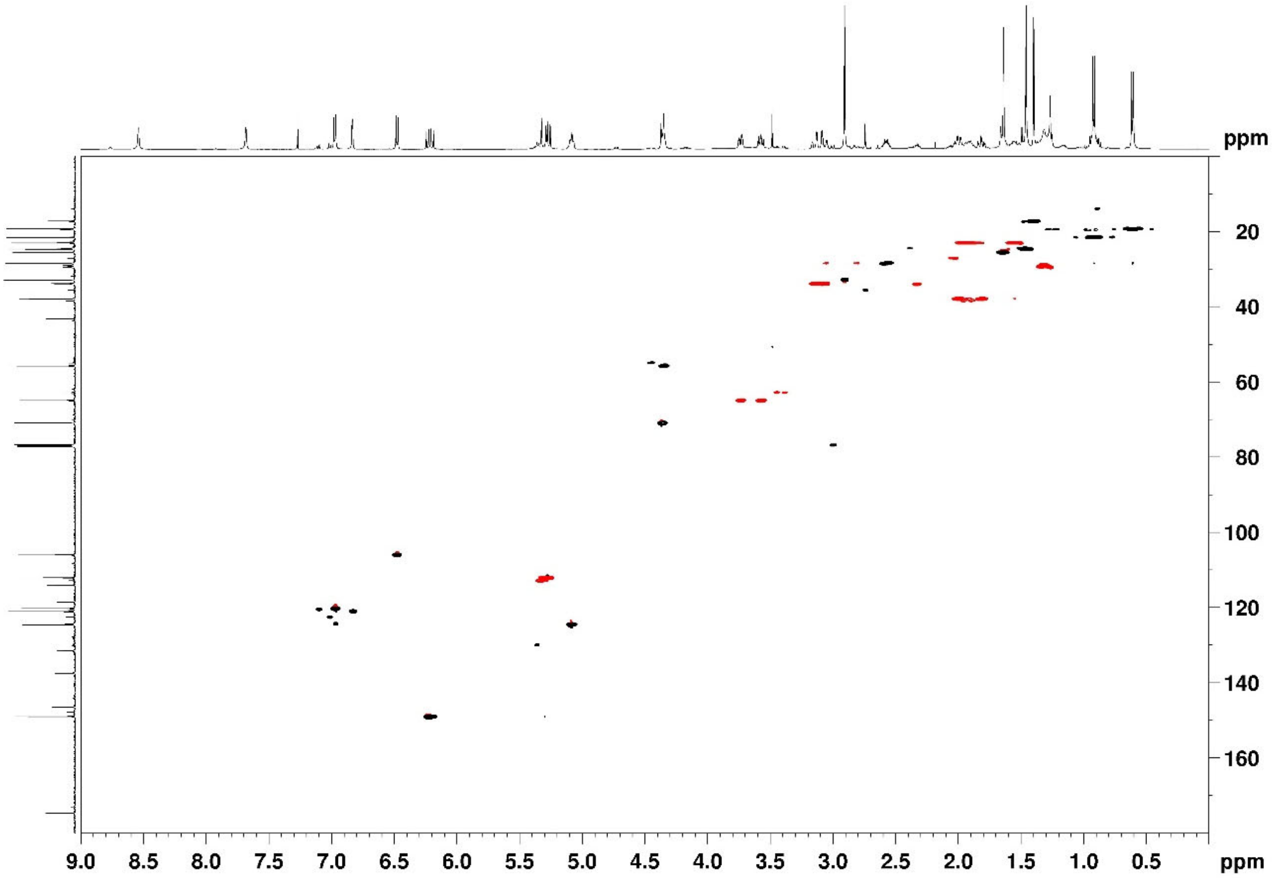

Fig. S10. HSQC spectrum correlation for teleocidin A.

Table S2. ^{1}H-NMR (500 MHz, $CDCl_3$) spectrum annotation for teleocidin A.

Position	Chemical shift (ppm)	Number of protons	Multiplicity	Coupling constant (Hz)
NH(1)	8.54, 8.77	1	2*s	
NH(10)	7.68	1	s	
$CDCl_3$	7.27		s	
6	6.97, 7.10	1	2*d	8.1, 7.9
2	6.83, 6.95	1	2*s	
5	6.48, 7.01	1	2*d	8.1, 7.9
21	6.21	1	dd	10.7, 17.8
22a	5.31	1	dd	1.1, 17.8
22b	5.27	1	dd	1.1, 10.7
25	5.08	1	m	
12	4.36	1	d	10.1
9	4.35, 4.46	1	2*m	
14-a	3.74	1	dd	3.4, 11.5
14-b	3.58	1	dd	8.1, 11.5
8a	3.15	1	d	17.2
8b	3.07	1	dd	3.7, 17.2
18	2.74, 2.91	3	2*s	
15	2.32, 2.58	1	2*m	
23a	2.00	1	m	
24a	1.91	1	m	
23b	1.82	1	m	
24b	1.55	1	m	
20	1.46, 1.49	3	2*s	
27, 28	1.40, 1.64, 1.66	6	3*s	
16, 17	0.61, 0.92	6	2*d	6.7

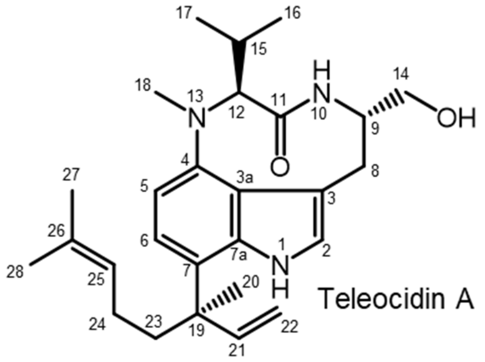

Table S3. ^{13}C-NMR (150 MHz, $CDCl_3$) spectrum annotation for teleocidin A.

Position	Chemical shift (ppm)	Number of carbons	Multiplicity
11	173.0, 174.7	1	2*s
21	147.8, 149.0	1	2*s
4	146.5	1	s
7a	137.5	1	s
26	131.4	1	s
25	124.6	1	s
2	121.0	1	s
7	121.0	1	s
6	120.2, 120.5	1	2*s
3a	118.5	1	s
3	114.0	1	s
22	112.1, 112.9	1	2*s
5	106.0, 122.5	1	2*s
$CDCl_3$			t
12	70.9	1	s
14	62.9, 65.0	1	2*s
9	55.0, 55.8	1	2*s
19	43.3, 43.7	1	2*s
23	38.0, 38.5	1	2*s
8	34.0, 34.4	1	2*s
18	33.0, 35.7	1	2*s
15	28.5	1	s
20	24.8	1	s
24	23.1	1	s
16, 17	19.4, 21.6	2	2*s
27, 28	17.3, 25.6	2	2*s

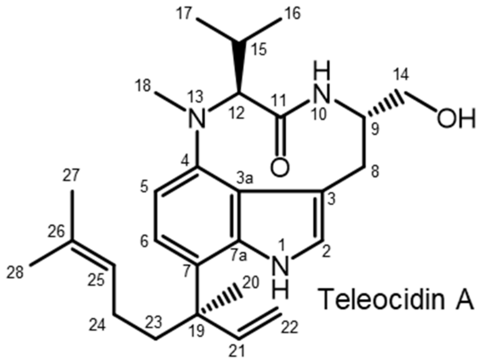

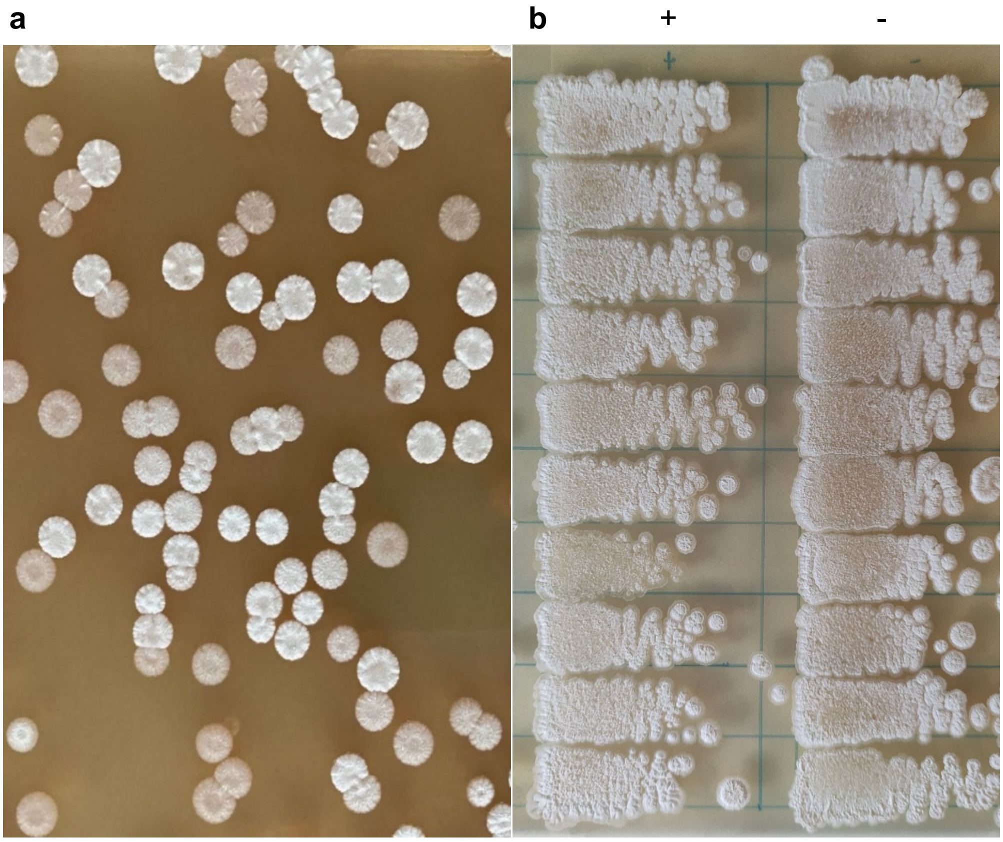

Fig. S11. a Morphological differences observed between the colonies the *S. clavuligerus* ATCC 27064 culture from the earliest ATCC vial (labeled “Dec. 15. 1986” by the ATCC), grown on ISP2 medium at 28°C for 14 days. One major distinction between the colonies was observed; some colonies have more pronounced aerial mycelium formation and earlier onset of sporulation (white colored) than others (skin colored). During experiment shown in Figs. 3 b and 3 c of the main text, we have observed that the distribution of the teleocidin producing phenotype (HPLC analysis) and genotype (PCR) within each of the two morphotypes was roughly the same as reported for the whole population (one third). **b** Random colonies from teleocidin-positive (+) and negative (-) subpopulation from experiment shown in Fig. 3 d of the main text. ISP2 plates were incubated at 28°C for 14 days. The differences in colony morphology were not observed in this case.

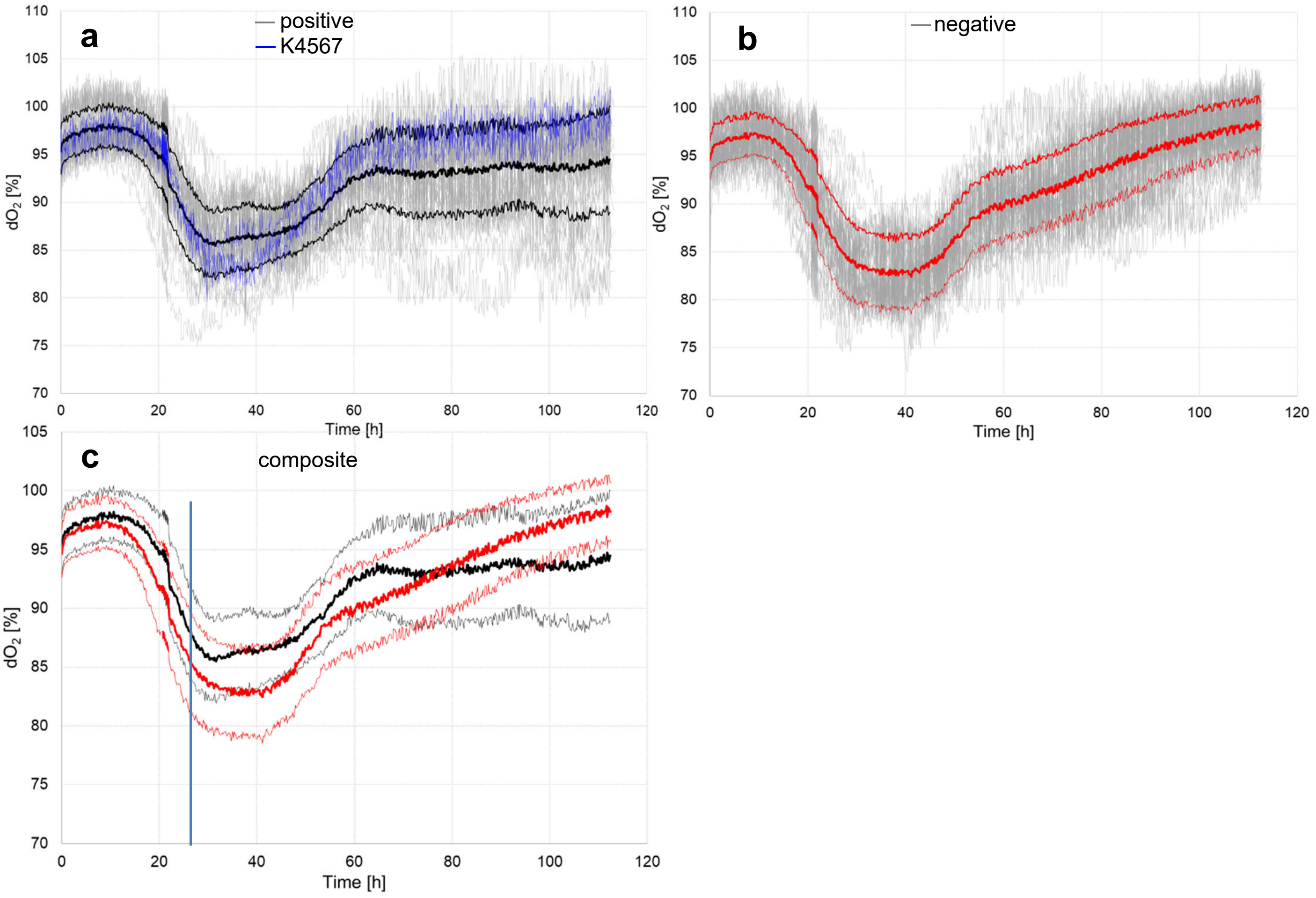

Fig. S12. Oxygen demand dynamics of the teleocidin-positive and teleocidin-negative sub-population of *S. clavuligerus* ATCC 27064 (labeled "Dec. 15. 1986" by the ATCC). 48 colonies of each subpopulation (refer to Fig. 3d and the main text) were cultivated in multiple 24-well microtiter plates equipped with calibrated dissolved oxygen (dO_2) sensors. Measurements were acquired in real-time, *in situ* and are shown for positive (**a**) and negative (**b**) subpopulations as thin grey lines. *S. clavuligerus* K4567 is shown as thin blue lines among the positive subpopulation (**a**). Average and standard error are shown in black for positive (**a**) and red for negative (**b**) subpopulations. Average (thick lines) and standard error (thin lines) for both subpopulations are overlaid on graph **c**. The transition between exponential and stationary phase is estimated with a blue line.

Table S4. Statistical evaluation for data in Figs. 3 c and 3 d.

Figure 3 c

Statistical analysis was performed using Mann-Whitney test

Column B	**negative**			
Column A	**positive**			
**P value**	<0,0001	**Difference between medians**		
Exact or approximate P value?	Exact	Median of column A	231	n=93
P value summary	****	Median of column B	163	n=275
Significantly different (P < 0.05)?	Yes	Difference: Actual	-68	
One- or two-tailed P value?	Two-tailed	Difference: Hodges-Lehmann	-59	
Sum of ranks in column A,B	26081, 41815			
Mann-Whitney U	3865			

Figure 3 d

Statistical analysis was perofrmed using repeated measured two-way ANOVA

**Two-way RM ANOVA**	Matching:	Across row			
Assume sphericity?	Yes				
Alpha	0,05				
Source of Variation	% of total variation	P value	Summary	Significant?	
Row Factor	17,99	0,685	ns	No	
Column Factor	0,0007598	0,9586	ns	No	
Subject	41,84	0,0085	**	Yes	
ANOVA table	SS	DF	MS	F(DFn, DFd)	P value
Row Factor	125022	47	2660	F(47, 96) = 0,8781	P=0,6850
Column Factor	5,281	1	5,281	F(1, 143) = 0,002704	P=0,9586
Subject	290819	96	3029	F(96, 143) = 1,551	P=0,0085
Residual	279259	143	1953		
Difference between column means					
Mean of positive	233,4				
Mean of Negative	233,1				
Difference between means	0,2708				
SE of difference	5,208				
95% CI of difference	-10,02 to 10,57				
Data summary					
Number of columns (Column Factor)	2				
Number of rows (Row Factor)	48				
Number of subjects (Subject)	144				
Number of missing values	0				

Table S5. Statistical evaluation for data in Fig. 4 c.

Figure 4 c

Statistical analysis was performed using one-way ANOVA with Tuckey's multiple comparison for each time point separately.

**24h**		Alpha	0,05							
**ANOVA summary**		**Tukey's multiple comparisons test**								
F	3,12200		Mean Diff	95,00% CI of diff	Below threshold?	Summary	Adjust. P Value			
P value	0,05210	K4567 vs. DtleA	0,4167	-3,595 to 4,429	No	ns	0,9661	A-B		
P value summary	ns	K4567 vs. D138kb	5,417	-0,01559 to 10,85	No	ns	0,0508	A-C		
Significant diff. among		DtleA vs. D138kb	5	-0,4323 to 10,43	No	ns	0,0771	B-C		
means (P < 0.05)?	No	Test details	Mean 1	Mean 2	Mean Diff	SE of diff	n1	n2	q	DF
R squared	0,10370	K4567 vs. DtleA	5,417	5	0,4167	1,665	24	24	0,354	54
		K4567 vs. D138kb	5,417	0	5,417	2,254	24	9	3,398	54
		DtleA vs. D138kb	5	0	5	2,254	24	9	3,137	54
**48h**		Alpha	0,05							
**ANOVA summary**		**Tukey's multiple comparisons test**								
F	0,62260		Mean Diff	95,00% CI of diff	Below threshold?	Summary	Adjust. P Value			
P value	0,54040	K4567 vs. DtleA	16,67	-20,45 to 53,79	No	ns	0,5292	A-B		
P value summary	ns	K4567 vs. D138kb	13,61	-36,65 to 63,87	No	ns	0,7917	A-C		
Significant diff. among		DtleA vs. D138kb	-3,056	-53,31 to 47,20	No	ns	0,9882	B-C		
means (P < 0.05)?	No	Test details	Mean 1	Mean 2	Mean Diff	SE of diff	n1	n2	q	DF
R squared	0,02254	K4567 vs. DtleA	115,8	99,17	16,67	15,4	24	24	1,53	54
		K4567 vs. D138kb	115,8	102,2	13,61	20,85	24	9	0,923	54
		DtleA vs. D138kb	99,17	102,2	-3,056	20,85	24	9	0,207	54
**72h**		Alpha	0,05							
**ANOVA summary**		**Tukey's multiple comparisons test**								
F	0,24890		Mean Diff	95,00% CI of diff	Below threshold?	Summary	Adjust. P Value			
P value	0,78060	K4567 vs. DtleA	11,97	-31,85 to 55,80	No	ns	0,7882	A-B		
P value summary	ns	K4567 vs. D138kb	11,84	-47,22 to 70,89	No	ns	0,8795	A-C		
Significant diff. among		DtleA vs. D138kb	-0,1389	-58,85 to 58,57	No	ns	0,9999	B-C		
means (P < 0.05)?	No	Test details	Mean 1	Mean 2	Mean Diff	SE of diff	n1	n2	q	DF
R squared	0,00930	K4567 vs. DtleA	297,4	285,4	11,97	18,18	23	24	0,932	53
		K4567 vs. D138kb	297,4	285,6	11,84	24,49	23	9	0,683	53
		DtleA vs. D138kb	285,4	285,6	-0,1389	24,35	24	9	0,008	53
**96h**		Alpha	0,05							
**ANOVA summary**		**Tukey's multiple comparisons test**								
F	0,30010		Mean Diff	95,00% CI of diff	Below threshold?	Summary	Adjust. P Value			
P value	0,74210	K4567 vs. DtleA	-9,702	-53,53 to 34,13	No	ns	0,8548	A-B		
P value summary	ns	K4567 vs. D138kb	-17,62	-76,06 to 40,82	No	ns	0,7482	A-C		
Significant diff. among		DtleA vs. D138kb	-7,917	-65,25 to 49,42	No	ns	0,9407	B-C		
means (P < 0.05)?	No	Test details	Mean 1	Mean 2	Mean Diff	SE of diff	n1	n2	q	DF
R squared	0,01163	K4567 vs. DtleA	425,7	435,4	-9,702	18,16	21	24	0,756	51
		K4567 vs. D138kb	425,7	443,3	-17,62	24,21	21	9	1,029	51
		DtleA vs. D138kb	435,4	443,3	-7,917	23,75	24	9	0,471	51
**120h**		Alpha	0,05							
**ANOVA summary**		**Tukey's multiple comparisons test**								
F	0,60490		Mean Diff	95,00% CI of diff	Below threshold?	Summary	Adjust. P Value			
P value	0,55000	K4567 vs. DtleA	-20,83	-74,41 to 32,75	No	ns	0,6186	A-B		
P value summary	ns	K4567 vs. D138kb	4,444	-67,00 to 75,88	No	ns	0,9876	A-C		
Significant diff. among		DtleA vs. D138kb	25,28	-44,81 to 95,37	No	ns	0,6611	B-C		
means (P < 0.05)?	No	Test details	Mean 1	Mean 2	Mean Diff	SE of diff	n1	n2	q	DF
R squared	0,02317	K4567 vs. DtleA	550	570,8	-20,83	22,2	21	24	1,327	51
		K4567 vs. D138kb	550	545,6	4,444	29,59	21	9	0,212	51
		DtleA vs. D138kb	570,8	545,6	25,28	29,03	24	9	1,231	51

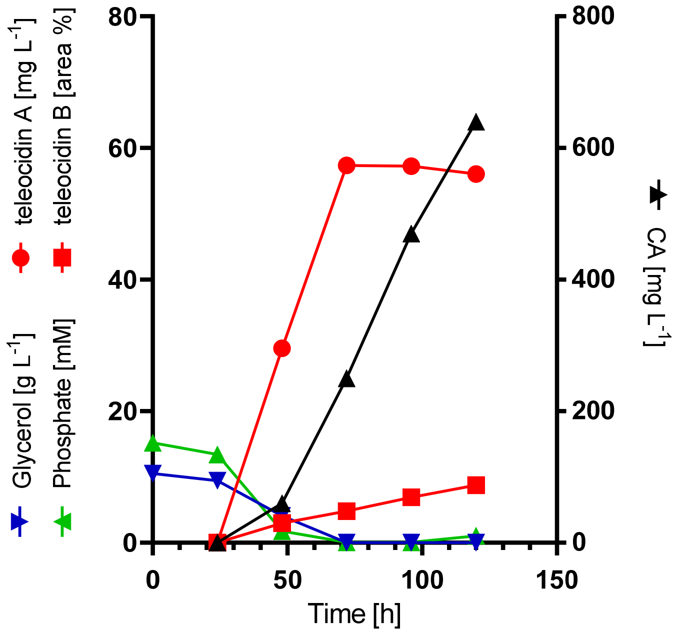

Fig. S13. Production of CA and Teleocidins, as well as glycerol and phosphate consumption of a typical shake flask culture of *S. clavuligerus* K4567.

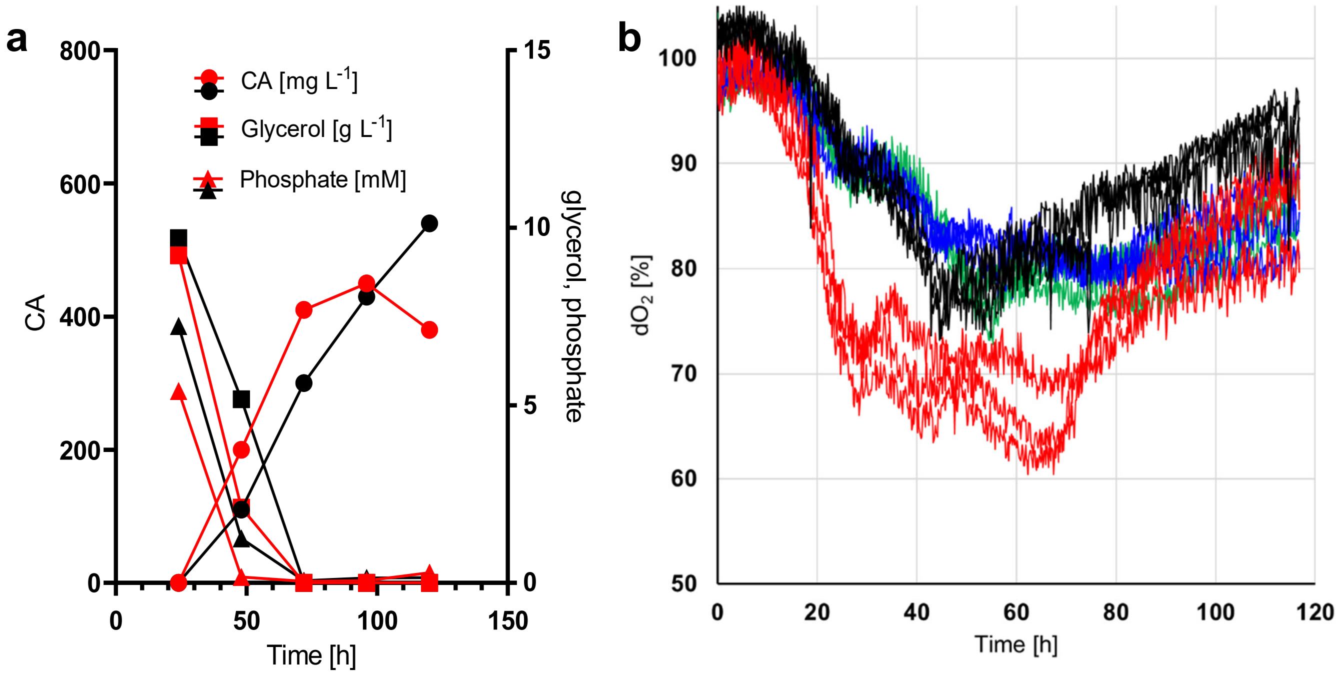

Fig. S14. An example of the influence of inoculum size on nutrient consumption and secondary metabolite accumulation dynamics in cultures for *S. clavuligerus* strains in this study. **a** Exemplary *S. clavuligerus* K4567 Δ138kb mutant shake flask cultures are shown where the seed phase was inoculated with either 1 cm^2 patch (**black**) or 4 cm^2 patch (**red**) of agar plate culture. **b** Oxygen demand dynamics of *S. clavuligerus* K4567 (**blue**) compared to the Δ*tleA* (**green**) and Δ138kb (**red**) mutants. Cultures were cultivated in 24-well microtiter plates equipped with calibrated dissolved oxygen (dO_2) sensors. Measurements were acquired in real-time, *in situ* and are shown in triplicates for each strain. 1.5 mL cultures were inoculated with 30 µL of seed phase. *S. clavuligerus* K4567 Δ138kb seed phase was prepared in two variants as described above. The reduced inoculum variant is shown in **black**.
